# Effects of the interplay between topology and function of an integrated urban development on patterns of user movement

**DOI:** 10.1038/s41598-024-57475-3

**Published:** 2024-03-25

**Authors:** Ajaykumar Manivannan, Wei Chien Benny Chin, Srilalitha Gopalakrishnan, Daniel K. H. Wong, Thomas Schroepfer, Roland Bouffanais

**Affiliations:** 1 Department of Mechanical Engineering, University of Ottawa, Ottawa, K1N 6N5 Canada; 2 Department of Geography, National University of Singapore, 117568 Singapore, Singapore; 3 Future Cities Laboratory Global, Singapore-ETH Centre, 138602 Singapore, Singapore; 4 Architecture and Sustainable Design, Singapore University of Technology and Design, 487372 Singapore, Singapore; 5 Department of Computer Science & Global Studies Institute, University of Geneva, 1211 Geneva, Switzerland

**Keywords:** Mechanical engineering, Environmental economics

## Abstract

With the advent of distributed multi-sensory networks of devices, vast troves of real-time data can be gathered about our interactions with the built environment. These rich data sets can be mined to achieve improved and informed data-driven designs of buildings, neighborhoods, and potentially entire cities. Among those, integrated developments have the peculiarity of combining multiple functions within a compact space and, as such, behave as microcosms of a city that can help address the problem of urban sprawl and density. However, a general lack of data and framework about integrated developments hinders our ability to test design hypotheses about the complex interplay between heterogeneity in both space and function. Here, we apply a data-driven approach to analyze the joint influence of topology and function on user movement within a state-of-the-art integrated development in Singapore. Specifically, we leverage the network representation of the building and use movement data collected from 51 individuals over a month. We show evidence of correlation (40%) between the spatial network features and human movement at the building level. We are also able to quantify the relationship between the functional and spatial components of the integrated development through user movement. Previous studies have shown a 60% or higher correlation between the topology and human movement at the city or country scales. Our moderate correlation, therefore, implies that more factors influencing user movement are at play. The heterogeneity in the spatial function introduced trips with diverse origins and destinations. A further data-driven analysis integrating origins and destinations reveals both qualitative and quantitative means of studying the relationship between the built environment and the processes that take place in them.

## Introduction

The future of human settlements is projected to be urban with the ratio of the world population residing in cities expected to increase from 55%, as of today, to 68% in 2050^1^. Urban areas are responding to this unabated trend in mostly two non-mutually-exclusive ways: (1) with a horizontal growth according to the well-known urban sprawl model, and (2) with an increase in density that usually leads to vertical growth, typically when faced with land scarcity and/or strained transportation infrastructure.

As our understanding of cities and their relationship to the socio-economic parameters concerning the human population improves^2,3^, better city planning is not only expected, but is an imperative. As the impact of climate change is more visible and pressing, cities are also forced to take their environmental impact into account. Hence, future cities aim to focus on sustainable development along these three verticals—economics, social, and environment. One of the stated seventeen goals of the United Nations (UN) Sustainable Development Goals (SDG) for the year 2030 calls for cities to be inclusive, safe, resilient, and sustainable^4^.

As factors like social welfare (community happiness, health, inclusivity) and environmental sustainability (waste management, energy consumption, environmental impact) are brought to the forefront, future cities look for an integrated solution to these challenges. Cities are shown in some cases to be efficient producers of economic^3^, social^5^, and environmental^6,7^ benefits. So, it is no surprise, that some of the solutions developed by private and public sectors are to build microcosms of cities in urban areas known as *mixed-use developments*^8,9^.

Many definitions exist for mixed-use development, but it is commonly defined as a well-planned development that integrates more than one function—or specifically, more than one program as it is referred to by architects and urban planners (e.g., commercial and residential)^10^. They have been a fixture of both urban and sub-urban areas to address density, optimize land use, and are seen as an emerging trend in future cities^11^. We hereby refer to mixed-use developments that consist of commercial, residential, and community (public) facilities closely integrated with transportation networks as an integrated development. However, it is worth noting that the term ‘integrated development’ is not well defined. Our study uses the term ‘integrated’ to define both outward-integration (to the larger spaces such as a district) and inward-integration (e.g., multi-level and street-level connections among all spatial and functional parts of the development). Integrated developments show promise to be part of the solution to address the UN SDG goals. They have the potential to improve social interactions, reduce demand for transport and other infrastructures, and improve the safety and vitality of the community^12^. However, research on or related to mixed-use developments is sparse^10,12,13^.

Beyond better land-use planning for the built environment, city planners are adopting data-driven approaches for the design of the so-called ‘smart city’^14,15^. The smart city concept can tap the potential of distributed multi-sensory networks—including solutions based on the Internet of Things (IoT)—to measure various features of a city such as traffic, energy, waste, environment, and people^16^. These measurements constitute ‘big data’ for the city, which can be assimilated to improve the real-time efficiency of a range of operations and to build better cities for the future. A similar approach has been developed specifically at the building level—the so-called ‘smart building’ framework^17,18^.

Hence, data collected from human behavior in a given city or building is related to spatial and environmental measurements that can be harnessed to improve a range of features of the built environment such as walkability, ease of navigation, and convenience^19^. This has been traditionally performed for street networks from the city scale down to the neighborhood scale. It is also not uncommon to find studies that extend those concepts down to the building scale^20–22^ or even the floor level^23^. These studies generally represent the structure of the built environment as a network and the human behavior related to it as a dynamic process taking place in it. In Architecture, this network representation is often considered within the space syntax framework^24,25^, which has frequently been cited as being limited in scope due to its dual graph representation^26,27^. Urban researchers and geographers have adopted a more general network-theoretic approach to studying the urban system and its components using *network science*^28^. Over the last two decades, network science has grown explosively and imposed itself as a powerful abstraction that enables the representation and study of many types of systems as networks, and that across a wide range of disciplines (e.g., human, ecological, and robots)^29^. For details on the difference in approach between space syntax and other network representations of the built environment, we refer the reader to the work by Porta et al.^27^.

Currently, there is a lack of a general framework to analyze the effectiveness of integrated developments. Unlike a sprawling city or a mixed-use street, the integrated development introduces heterogeneity in space and function in a high-density built environment that is clearly more complex than single-use developments^30^. Another key challenge is the lack of appropriate data that can help test or evaluate a given design hypothesis for an integrated development. Evidence-based or data-driven design^31^ is a growing trend in urban development based on the premise that we cannot improve what we cannot measure. The same can be said at the integrated development level, hence pointing to the pressing need to gather data, process and analyze it.

Human movement in a building is commonly referred to as circulation. The study of circulation is an essential part of the design process in Architecture that aims to improve legibility, navigation, accessibility, safety, and optimal connectivity^20^. The relationship between human movement and the built environment (city^32^, street^33^, building^20,34^, or a floor^23^) has been extensively studied using space syntax and other network-theoretic methods^35–37^. This is done so using socio-spatial theories or based on movement data. However, movement data in integrated developments, or more broadly in mixed-use developments, are sparse. Chang^30^ collected movement data (adjacent and path) from 300 users in an integrated development (Barbican, London, U.K.) and a sprawling mixed-use development (South Bank, London, U.K.) based on visual observations to study individual route choice and movement behavior. In addition, Willis et al.^33^ collected movement data from 2613 participants in three mixed-use streets in the U.K. based on video-recorded observation to study pedestrian movement. These studies^30,33^ belong to the rare cases of works that analyzed movement data in mixed-use developments. It is apparent that the scant presence of adequate human movement data^38^ hinders our ability to test design hypotheses in mixed-used developments. Our study introduces movement data that tracked 51 participants over a long period of time (1–5 days) in a free-living environment with high temporal ($\sim \,1$ Hz) and spatial resolution ($\sim \,20$ m) within a new state-of-the-art integrated development in Singapore. Human Activity Recognition (HAR), such as tracking and identifying human movements precisely in a three-dimensional (3D) environment, requires a suite of sensors (e.g., barometer and IMU^39^) and involves complex data processing^40^. This study uses strategically placed Bluetooth beacons (environment) and mobile applications (peer) to finely track user movements.

We adopt a general network-theoretic approach similar to urban researchers and geographers^41^. We study network representations of factors in the built environment that are not embedded in space, i.e., besides choosing built environment fixtures as nodes and their adjacent pathways as edges, we represent the spatial functions and their relationships as a weighted network that is not necessarily embedded in space, and we also aggregate spatial components for abstract analysis^42^. This method is similar in essence to the approaches used to study other networks like social or ecological networks, where more abstraction of network components results in a better understanding of the network process and its system-level operation. This framework and methodology are especially relevant in studying integrated developments that introduce complexity in the relationship between humans, space, and function. As mentioned previously, this method is different from space syntax methods traditionally used in architecture. We refer the interested readers to work done by Zhang and Chiaradia^43^, for the application of space syntax methodology to understand three-dimensional mixed-use areas using user movement.

Hence, analyzing the factors that influence user movement in an integrated development pave the way towards a better understanding of the intricate relationship between human activity and the built environment. To improve the design of these developments, data-driven approaches have the potential to uncover all the factors affecting human movements such as the human aspect, the spatial aspect, location, time, and climate^19,44^. On the one hand, spatial factors include geometry, connectivity, visibility, spatial integration^34^, centrality^45^, intelligibility^20^, legibility, walkability^46^, and spatial function^23^. On the other hand, human factors include familiarity with the environment^30^, spatial cognition, user profile, and user preferences^33,47^. In this study, we focus on two spatial factors, namely topology and function. The term ‘function’ refers to space utilization i.e., primary use of space such as commercial, community, corridor, or vertical facilities.

In both space syntax studies and the general network-theoretic approach, the appropriate use of distance measures is an ongoing topic of research. The distance between two nodes in a network can be given by its topological (number of hops), Euclidean (straight line metric distance or distance along the network), angular (sum of deflection angles), or hybrid distance (combination of the above). Analyzing the road network of the UK using vehicular data, Serra and Hillier^48^ showed that angular distance is a better predictor of vehicular movement than Euclidean distance. However, the study also showed a very high correlation between the betweenness centrality measures when using angular and Euclidean distance at small spatial scales ($\sim \,2$ km). Similarly, Zhang et al.^49^ analyzed the road network of Shanghai, China, and found that angular and Euclidean distances are highly correlated (80–90%). Moreover, Zhang and Chiaradia^50^ studied the three-dimensional pedestrian network of Central, Hong Kong using topological, Euclidean, angular, and hybrid (Euclidean and Angular) distance, and showed that the betweenness centrality correlation (spatial measure and movement) is the same for all except the topological distance. The study however also shows that this is not the case for closeness centrality correlations. More research is required to understand the effect of studying the relationship between spatial measures and user movement using different distance measures. In addition, Cooper^51^ notes that users unfamiliar with a route tend to take the path with few angular turns. On the other hand, familiar users are prone to select routes that minimize the Euclidean distance. As the participants tracked in our study are residents, employees, and frequent users of the space, they are very effectively familiar with the building and its configuration. Therefore, the Euclidean distance has been selected as the natural distance metric.

It is worth noting that the Euclidean distance along the network is different from the classical Euclidean distance, where the latter is calculated by drawing a straight line between one node to another while ignoring the presence or absence of a direct physical connection between them. Our study thus uses the Euclidean distance along the network (a.k.a. routing distance) to reflect the realistic distance a user will traverse and in some cases, the network is purely analyzed by its topology. In addition, the choice of Euclidean distance along the network is appropriate for familiar users known to minimize the distance

We ask ourselves the following question: how do topology and function influence user movement/circulation in a complex integrated development? User movement data is collected in Kampung Admiralty (KA), Singapore’s first integrated public development that brings together a mix of public facilities (healthcare, gardens, and community spaces), commercial facilities (shops, banks, and restaurants), and a residential complex^52–54^. KA serves as our case study to demonstrate the promise of a data-driven approach in this framework and the power of a network representation to understand human-built environment relationships in a high-density complex environment. Our contribution to integrated development analysis is limited to KA’s inward-integration property while its outward-integration is only studied up to the multiple entrances that seamlessly integrate into the rest of the district. The space beyond KA’s entrances is out of the scope of this study. Our results can be summarized as follows: (1) we establish a clear quantitative relationship between building topology and user movement, (2) we uncover the details of the interplay between topology and function in influencing user movement, and (3) we quantify the magnitude of movement flow within and between the spatial and functional components of the integrated development.

## Results

The study aims to understand the impact of building topology and function on the user movement within. While the KA spatial network is an abstraction of the building topology, the user movement can be studied as flows across this connected network. To uncover hidden patterns of user movement, we aggregate the movement data according to specific floor groups and spatial functions (refer “Methods” section).

The first section quantifies the relationship between network topology and user movement. The second section looks at the interplay between connectivity and function through user movement data and the underlying spatial network. The third section analyzes the homophilic and heterophilic movement flow across the vertical components of the building and spatial functions and explores the impact of their relationship on user movement. In this last section, we also use the origin-destination matrix to understand the influence of topology and function on the beginning and end of each trip taken by the user and finally calculate the node entropy to look at the diversity of the movement flow across the spatial and functional groups (refer “Methods” section).

### Influence of spatial network topology on user movement

As a first step, we attempt to define a clear quantitative relationship between the topology and user movement.

Various centrality measures can determine a particular node’s importance in a network. The description of the selected node centrality measures and their relevance to the built environment are explicitly listed in Table 1. We calculated the Spearman’s rank correlation coefficient between node centralities (in-degree, weighted closeness, and weighted betweenness) and movement flow to understand the relationship between the topological network properties and the network process—i.e., the user movement flow. A moderate correlation ($\sim \,0.4$) is observed between all of the topological properties and the movement flow with statistical significance ($\text {p-value} \le 0.001$) (Table 2). This shows that the network topology itself has a moderate influence on how people move about throughout KA. Previously, these results have been observed for street networks and other transportation networks^55–58^. At the building level, these results are consistent with other works where building configuration is quantitatively shown to influence user movement^34^. However, an explicit quantification of the magnitude of this topological influence, as shown here at this scale, is still rare^59^. As we break down the KA spatial network and examine its sub-graphs, the correlation values change for different floor groups. Level-1 and L-2 have a high correlation (0.5–0.8) between the given topological properties and movement flows, while car-parks show low-to-zero correlation (Table 2).

The topology’s moderate influence means that many other parameters drive the movement flows, including function, user preferences, and other properties that are not studied in this work such as weather conditions, spatial enclosure (partly indoor/outdoor), spatial cognition^60^, and facilities (e.g., presence of sitting places)^59^.

**Table 1:** Node centrality measures and their relevance to studying the built environment.

Node centrality	Description	Relevance to built environment
In-degree	Number of incoming connections to the node	A node with high in-degree centrality has many incoming connections and hence it is said to have high connectivity locally
Closeness	Average distance of a node from all other nodes	A node with high closeness centrality is said to be the optimal node (to minimize distance) to start to reach all other nodes. For example, designers can place an entry point or key zone with high closeness centrality for optimal reachability to/from all other nodes
Betweenness	The extent to which a node lies on the shortest path between other node pairs	A node with high betweenness centrality is said to be central to all the traffic flowing between other node pairs. For example, if a designer wants a key zone to be placed in such a way that most people walk through it when traveling between any two locations, the node can be designed to have high betweenness centrality

**Table 2:** Spearman’s rank correlation coefficient results between topological network centralities and mobility (incoming flow) for all and each floor-group.

Floor-groups	In-degree	Closeness (weighted)	Betweenness (weighted)
All nodes	0.402***	0.387***	0.391***
Car-parks	0.094	0.055	0.14
Level-1	0.571**	0.337	0.655***
Level-2	0.541*	0.782***	0.595*
Level-6	0.37	0.203	0.216
Rooftop garden	− 0.023	0.486	0.607*
Residential	0.438*	0.334	0.307

The closeness and betweenness centralities are weighted by routing distance.

***p-value $\le$ 0.001; **p-value $\le$ 0.01 ; *p-value $\le$ 0.05.

### Interplay between connectivity and function

In the previous section, we established a quantitative relationship between topology and user movement. In this section, we analyze and demonstrate the influence of topology and function broadly. When a user moves from one location to another in an integrated development, she moves across various spaces with particular functions. The user is also moving along the designed topology of the built environment across the horizontal and vertical directions. Does a user move from one space to another due to spatial function, or does better connectivity allow for it? The complex interplay between these factors influencing the movement volume is illustrated and analyzed. This section takes both a qualitative (based on on-site observation and knowledge of the building) and a quantitative (aggregated movement data and weighted spatial network visualization) look at the results.

**Figure 1:**
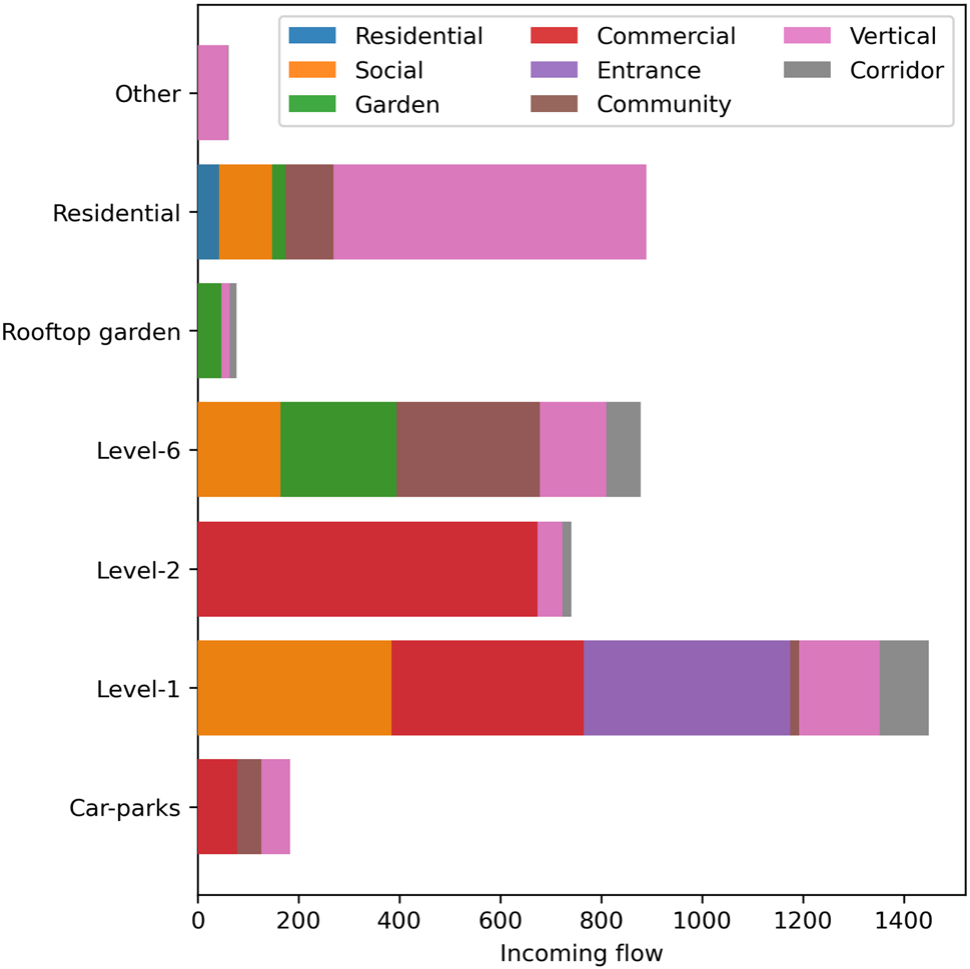
Distribution of inflow by floor-group and function categories.

The number of movement inflow for a given node in the KA spatial network is studied according to floor group and further sub-divided by function type as shown in Fig. 1. The aggregated distribution shows rich movement flow across the vertical and horizontal dimensions. Level-1 (ground floor) has the highest inflow due to its accessibility from the ground (entrance), large open plaza (social space), and its contribution as the main commercial street with shops and restaurants (commercial). This particular diversity of function is clearly reflected in the significant movement flows in the above-said function categories. Level-6 connects the residential and commercial components of KA and sees the second most movement inflow, similar in magnitude to the entire residential building that has seven floors. Residential movement is overwhelmingly in the vertical dimension as there is little horizontal spread on residential floors. This is followed by Level-2 that only consists of food and beverage stalls with accompanying sitting areas, which nevertheless see a significant movement flow. The car-parks (Basements 1 and 2) and rooftop gardens (Level 8 and 9) form the two corners of KA’s vertical structure, and unsurprisingly they exhibit little movement flow. The car-parks consist of commercial spaces such as supermarkets and banks. The majority of the user movements within those floors is associated with these commercial spaces.

**Figure 2:**
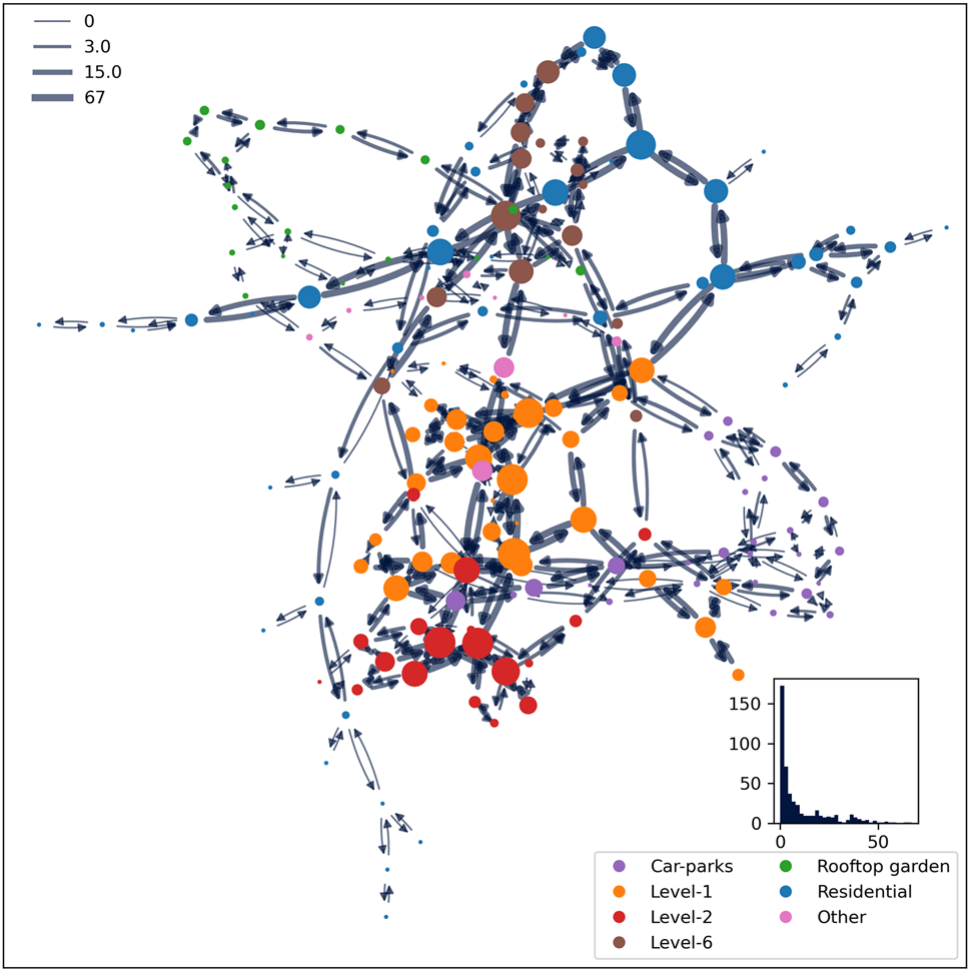
KA spatial network. Nodes are colored by floor group and sized by movement inflow. Directed edges are sized by the number of movement flows in respective directions. Bottom right plot: Histogram of inflow count. Top left: Legend indicating flow size for minimum, 50th percentile, 75th percentile, and maximum values (edges sized by taking the logarithm of flow count for better contrast in visualization).

The usage of garden spaces on Level-6 can be unavoidable if a user needs to cross between the residential and the commercial tower on that floor. This is, however, not the situation for the rooftop garden where the user movement indicates a deliberate use of garden space. In fact, the users have vertically traveled to the top floor of the building with the sole intention to do so. The spatial network of KA is shown in Fig. 2 where the nodes are colored by floor group and sized by inflow. The spatial network tapers at the two spatial ends of the KA building. Both connectivity and function drive the movement distribution between those two ends (car-parks and rooftop gardens). However, the Level-6 connection between the two mid-rise towers has allowed for higher movement flow, where connectivity has influenced better movement flow than otherwise possible with floor function alone. This is seen by the slight movement flow on Level-7, which has a similar function space grouped under the category ‘Other’ in Fig. 1.

**Figure 3:**
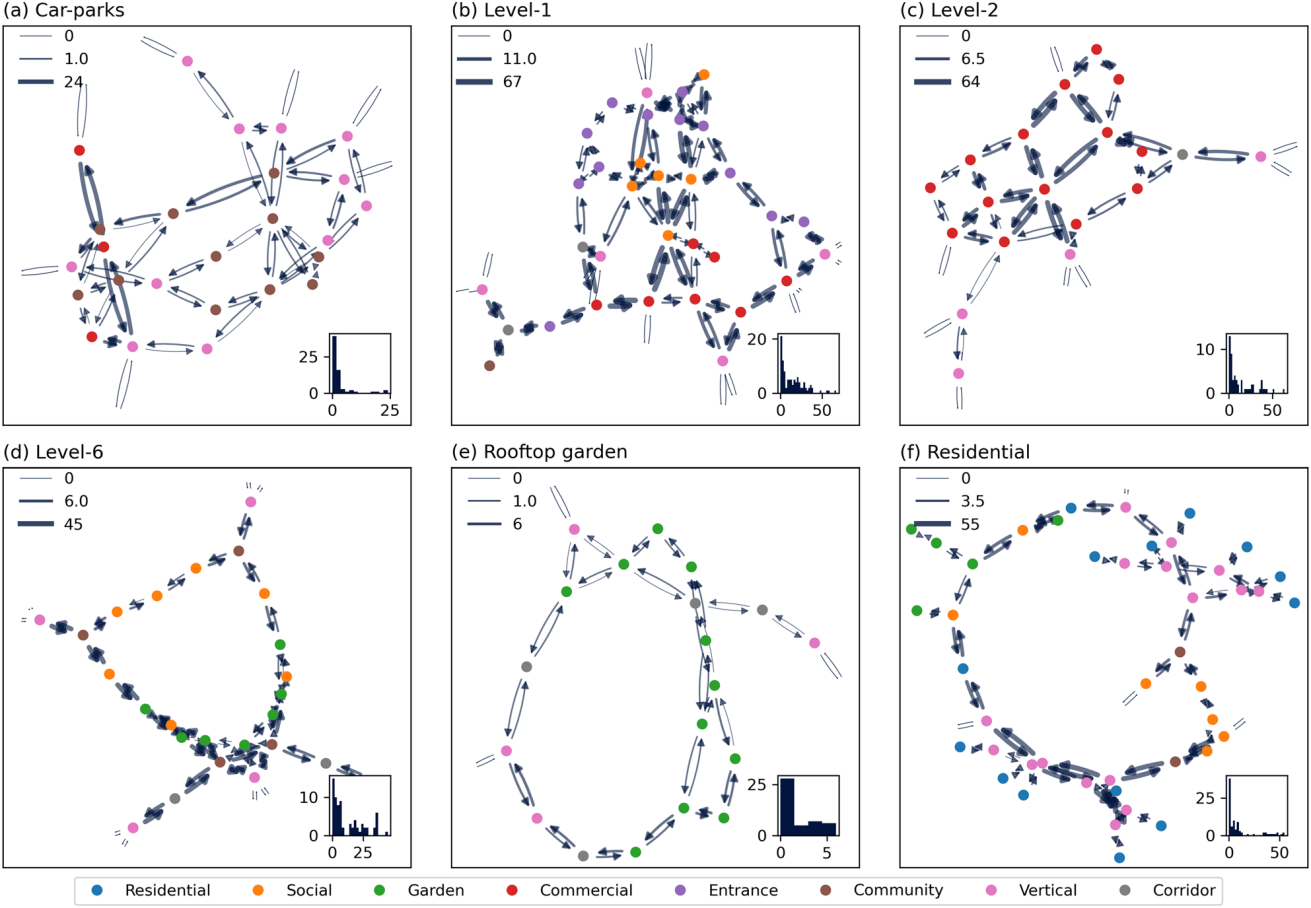
Subgraphs of the spatial network. (**a**) Car-parks (B1 and B2), (**b**) L1, (**c**) L2 (food court), (**d**) L6, (**e**) rooftop garden (L8 and L9), and (**f**) the two residential towers. The colors indicate function categories. Bottom right plot: Histogram of flow count. Top left: Legend indicating flow size for minimum, 50th percentile, and maximum values (edges sized by taking the logarithm of flow count for better contrast in visualization).

The movement patterns in the six-floor groups are analyzed and presented in this section (Fig. 3). The vertical streets (lifts, escalators, and stairs) form the KA spatial network sub-graphs cornerstones. They serve as the main access points for elevated or basement floors. The movement distribution is concentrated at some point of the network sub-graph for car-parks, Level-2, Level-6, and residential buildings. However, this concentration is not always a property of the network itself, i.e., only some nodes of these sub-graphs have mobility flow proportional to the node degree, such as for instance the center point of Level-1 and Level-2. In some cases, the movements are clearly influenced by function, e.g., commercial nodes of car-parks and the commercial side of Level-6. Nevertheless, there is significant movement flow at multiple points of the sub-graphs showing good circulation throughout KA in most cases.

In summary, this section looked at the aggregated distribution of movement data and weighted spatial network visualization to illustrate the influence of connectivity and function on user movement. Qualitatively, it can be inferred from the data analysis that both factors show varying degrees of influence.

### Homophilic and heterophilic movement flow

So far, we have shown evidence of the impact of topology and function on user movement. In this section, we look at this influence in more detail and in a more quantitative way. How intense is the movement flow within and between the spatial and functional components of the integrated development? We specifically aim to study the influence of the relationship between the defined spatial and functional groups on user movement. This is done by studying the aggregated movement data based on two types of movement data—adjacent movement (studied so far) and origin-destination of a trip.

#### Adjacent movement

The spatial network is aggregated by floor-group and functional-group. Table 3 shows the proportion of movement within (internal) and between (external) the six floor-groups and eight function categories. The internal flows (homophilic) account for ${80}{\%}\,\hbox {to}\,{90}{\%}$ of the nodes’ movement in the floor-group; i.e., only ${10}{\%}\,\hbox {to}\,{20}{\%}$ of the flows left each floor-group. Many of the function groups have the majority of the movement flowing externally. This may be because they are also physically scattered in space. An exception to this is the vertical and commercial function spaces. These groups are physically clustered and interconnected, thus allowing a higher proportion of the movement flow to remain internal.

**Table 3:** The proportion of internal flows and external flows for floor-groups and function categories..

Grouping	Category	Total	Internal	External
Floor-group	Car-parks	236	0.78	0.22
Floor-group	Level-1	1638	0.89	0.11
Floor-group	Level-2	877	0.84	0.16
Floor-group	Level-6	693	0.86	0.14
Floor-group	Rooftop garden	85	0.91	0.09
Floor-group	Residential	986	0.90	0.10
Function category	Entrance	402	0.50	0.50
Function category	Social	586	0.33	0.67
Function category	Commercial	1277	0.80	0.20
Function category	Community	351	0.18	0.82
Function category	Garden	306	0.34	0.66
Function category	Residential	42	0.00	1.00
Function category	Vertical	1496	0.72	0.28
Function category	Corridor	195	0.01	0.99

‘Residential’ in the Floor-group refers to both residential towers, while ‘Residential’ in the function categories denotes the individual apartments of the two residential towers that exclude vertical transportation and corridors.

**Figure 4:**
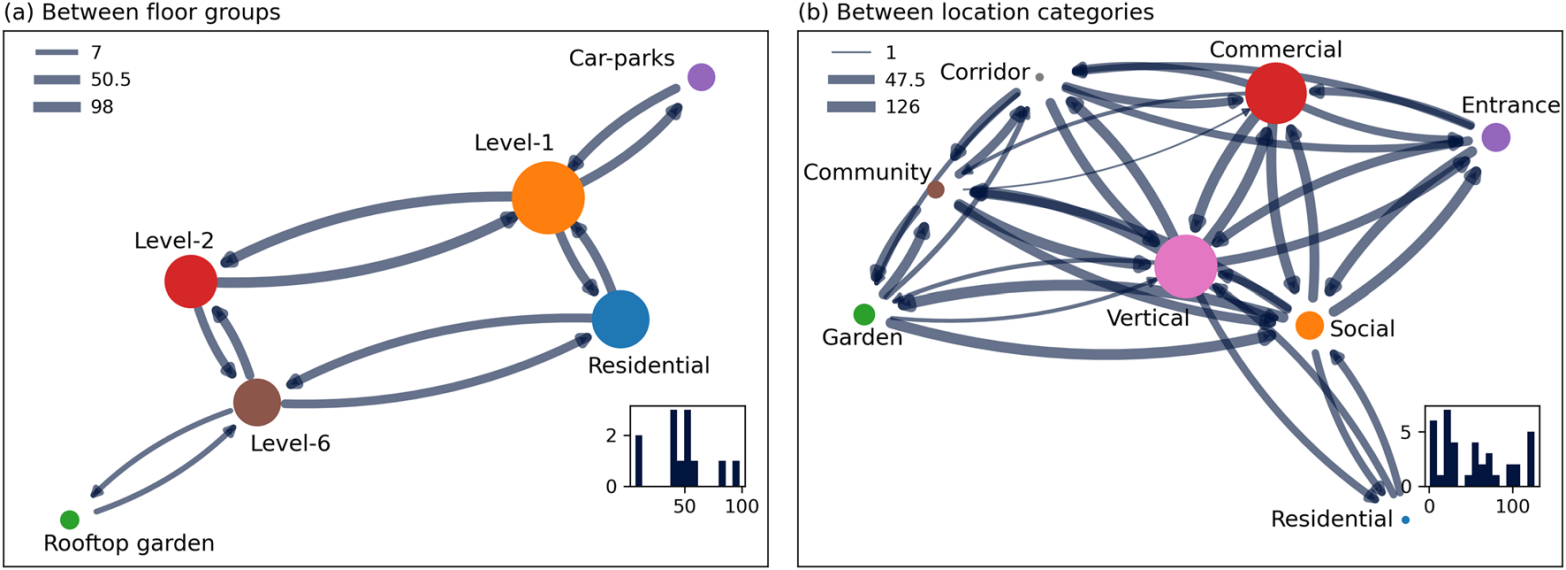
Networks of aggregated flow. (**a**) By floor-groups and (**b**) by function categories. The node sizes indicate internal flows (self-loop). Bottom right plot: Histogram of inflow count. Top left: Legend indicating flow size for minimum, 50th percentile, and maximum values (edges sized by taking the logarithm of flow count for better contrast in visualization).

Figure 4 presents the external movement flow of people by means of the widths of edges, and the size of internal flows is presented as the size of nodes. The external flow across the floor groups is more or less equal in volume between L-1, L-2, L-6, Residential, and the car-parks (Fig. 4a). However, the internal flow is highest on L-1, followed by the residential region and L-2. The lowest internal user movements are seen, not surprisingly, in the car-parks and rooftop garden. This is also clearly seen in Table 3. The external flows between different functional groups are more or less equal. An exception is a low movement between the community and commercial spaces. This only indicates that those two different functions are mostly physically separated, and hence only a few adjacent movements were possible. The residential units and corridor have zero internal movements because they are physically separated, and their sub-components are only connected through vertical function spaces like lift lobbies. The vertical spaces are the central function space connected to all other spatial functions, thus driving the majority of the external movements. Loosely speaking, these lift lobbies operate like hubs in KA. It is worth noting that the mobility data is not studied by hour of the day, weekday, or weekend due to the small sampling size in such temporal divisions. Such intricate analysis, however, could enrich this study by showing temporal patterns of human movement and its relationship to the built environment (see “Discussion” section for more details on the limitations of our study, including the small size of the representative population).

The vertical streets are one of the main pathways in KA integrated development. However, the high internal flow in commercial areas also indicates that the movement is heavily 3-dimensional. These results on user movement flow highlight the intricate relationship between functional and spatial units of the groups, and reveals the key players driving those movements.

#### Origin and destination of a trip

The previous section dealt with analysis of the homophilic and heterophilic movement flows. These results are limited since they are based on using adjacent movement data. Specifically, they ignore the two most important parts of user movement, namely the origin and destination of each trip. By studying the origin-destination matrix and its network representation, we aim to deepen our understanding of the relationship that exists across the vertical components of the building and spatial functions. The start and end of a trip bring the components of these groups closer in context as we see the user movement in full circle, and thereby sheds light on the destination preference of a user.

**Figure 5:**
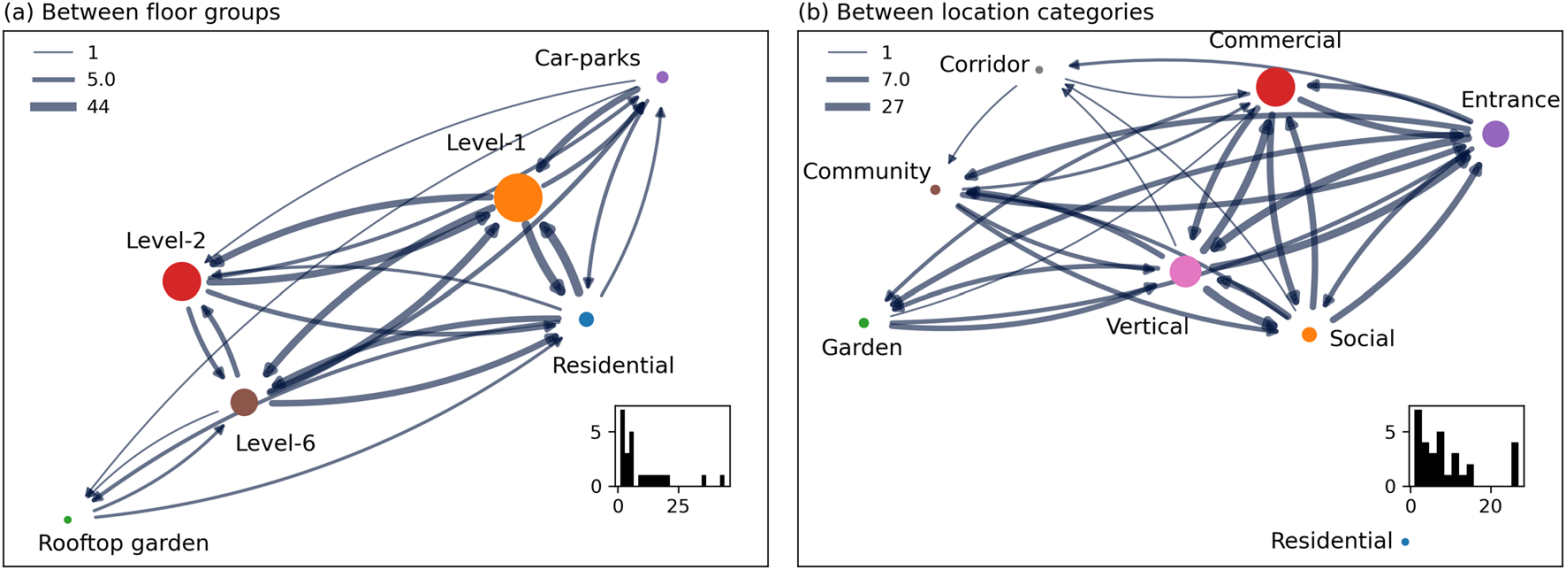
Networks of aggregated flow based on origin and destination of a path. (**a**) By floor-groups and (**b**) by function categories. The node sizes indicate internal flows (self-loop). Bottom right plot: Histogram of inflow count. Top left: Legend indicating flow size for minimum, 50th percentile, and maximum values (edges sized by taking the logarithm of flow count for better contrast in visualization).

**Figure 6:**
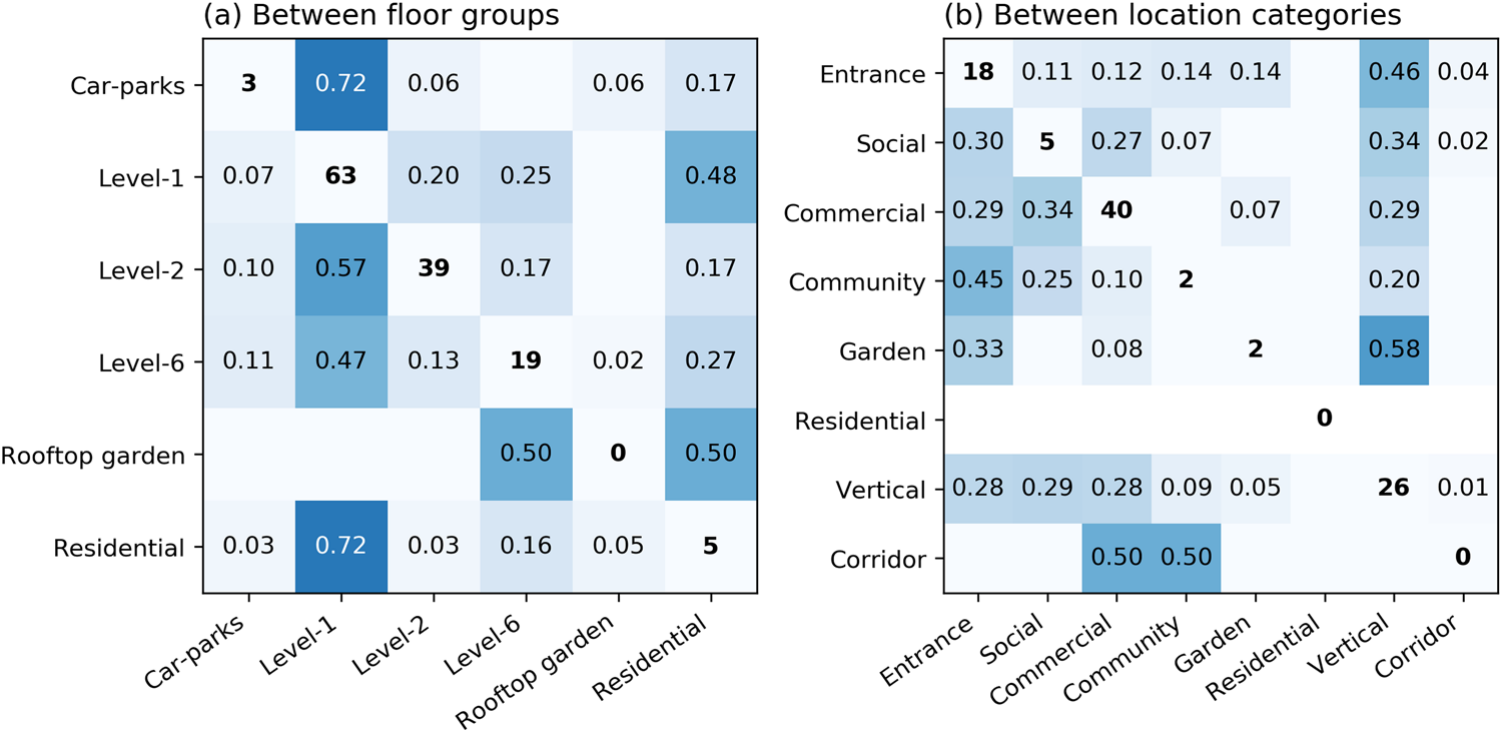
Row normalized origin-destination matrix. (**a**) Aggregated floor-groups network, and (**b**) aggregated function categories network. Reading the OD matrix: The diagonal values indicate the number of internal flows within each group. The row normalized values indicate the proportion of flows from one origin to every destination. For example, 0.72 in the first row indicates that 72% of movement originating in car parks ended at Level 1..

Figure 5 shows aggregated network graph with each directed edge indicating an origin and destination of a trip and its size denoting the volume of trips between them. The size of the nodes indicates the number of trips whose origin and destination are within the same node. Figure 6 shows the Origin–Destination (O–D) matrix with the diagonals denoting the internal trips. Most trips have originated or ended at level-1 (see Fig. 5a). The largest destination of trips originating from car parks is level-1, but the users have also reached level-2, residents, and rooftop garden (see Fig. 6a). The trips starting from the residential building have also majorly ended at level-1 and have reached every other floor. The large volume of users departing to and from the rooftop garden is from the residential building. As already mentioned, the residential units and corridor have zero internal movements because they are physically separated, and their sub-components are only connected through vertical function spaces like lift lobbies. This is clearly visible in Figs. 5b and 6b. The Level-1’s connectivity to the rest of the neighborhood and various function spaces allows most of the trips to start and end in level-1 as shown in Fig. 5a. Nevertheless, the edges between the vertically discrete spaces show that KA integrated development encourages a large volume of trips (and thus destination) within.

Nearly 50% of the trips starting at the entrances reach the vertical spaces, thus showing that those trips are eventually destined for elevated spaces (see Fig. 6b). The trips starting at entrances and vertical spaces see the most diverse destinations. Trips with origin and destination within the same function spaces are seen primarily in commercial and vertical spaces. This is similar to the results seen in the previous section using adjacent movement data. Similar to the floor group, the aggregated function group network is highly connected, thereby indicating trips with diverse origin and destination within KA.

Vertical nodes are not typically considered as an origin or destination but as transition nodes. However, they are present here as origin and destination due to the fact that—(1) vertical facilities are numerous and placed very close to several entrances of KA, and (2) exiting vertical nodes on higher floors can lead to private locations like residences that are not monitored. Considering vertical nodes as part of the O–D matrix enriches our three-dimensional study of mobility and provides an opportunity to understand the role of vertical nodes in facilitating trips within KA. Please refer to Supplementary Fig. S1 which features the O–D matrix with the vertical node removed.

#### Diversity of movement flow

Table 4 shows the normalized outgoing and incoming entropy, which respectively represent the diversity of outflows and inflows. A high value of outgoing entropy ($\text {H}^{\text {out}}\simeq \,1$) indicates evenly distributed outflows to all other nodes, whereas a low outgoing entropy ($\text {H}^{\text {out}}\simeq \, 0$) indicates the outflows were all going to the same destination. The highest flow diversity is seen in the outflow of Level-6 and inflow of car-parks and Level-1. Most other floors have low to moderate diversity of trip origin and destination. The car-parks have a moderate outgoing entropy and high incoming entropy, suggesting that the incoming trips have started from diverse locations while the diversity in the destination is low. The rooftop garden has the lowest outgoing entropy, thus indicating trips with few destination choices. The highest trip diversity is seen at the entrance and vertical spaces, as previously shown in the O–D matrix (see Fig. 6).

**Table 4:** Normalized outgoing entropy ($\text {H}^{\text {out}}$) and incoming entropy ($\text {H}^{\text {in}}$) of the floor-group and function categories based on origin and destination of a path.

Floor-group	$\text {H}^{\text {out}}$	$\text {H}^{\text {in}}$	Function category	$\text {H}^{\text {out}}$	$\text {H}^{\text {in}}$
Car-parks	0.477	0.738	Entrance	0.732	0.691
Level-1	0.671	0.709	Social	0.649	0562
Level-2	0.641	0.547	Commercial	0.614	0.600
Level-6	0.729	0.629	Community	0.605	0.561
Rooftop garden	0.387	0.530	Garden	0.427	0.492
Residential	0.505	0.615	Residential	0	0
			Vertical	0.716	0.690
			Corridor	0.333	0.500

The homophilic and heterophilic analyzes of movement flow show a strong movement within floors and function groups. By looking at the weighted spatial network at an aggregated level and considering adjacent movement and trip data, we quantified the influence of topology and function on the user movement.

## Discussion

This study is aimed at identifying and understanding the factors that influence user movement in an integrated development like KA, where residential, commercial, and public facilities are distributed in a vertical, high-density built environment. We specifically focused on two factors—topology (built environment layout and spatial network structure) and function. With Kampung Admiralty as a case study, we show that network-theoretic methods offer a powerful way of looking at data-driven designs at the building scale by associating the network process (user movement) with the network structure (built environment layout). We asked and answered specifically the following three research questions: (1) “What is the quantitative relationship between topology and user movement?”, (2) “Does a user move from one space to another due to spatial function, or does better connectivity allow it?”, and (3) “How intense is the movement flow within and between the spatial and functional components of the integrated development?”.

First, our results reveal a $\sim \,40$% correlation between the centrality measures of the KA spatial network and user movement, thereby indicating that the distribution of flows followed the topological network structure to a moderate level. Previous studies at the country- and city-scale suggested that the correlation between the daily human movement patterns and network metrics was higher at about 60%^55,56^. Urban planners and designers can benefit from an accurate and quantitative understanding of what drives people to move and spend time in locations. These are already widely studied in human mobility studies at the city scale for optimizing the allocation of facilities and efficient design of the built environment^61^. The question of which factors contribute to what proportion of movement can precisely guide the designers to focus their effort on factors that are proven to work. More research and data-driven post-occupancy studies are required to study the precise influence of all factors that affect user movement at different spatial scales, to understand both universal and subjective behaviors (e.g., design-dependent, and population-dependent).

Second, the analysis of the KA spatial network and its sub-graphs show a varying degree of intertwining influence by topology and function on user movement. Our results indicate that a user is driven to specific areas due to spatial function. However, the route choice of the user is influenced by connectivity. Traditionally in planar studies, route choices can be influenced by the shortest or the fastest route. In our case study, the user preferences for the route are often influenced by spatial functions along the route like garden spaces and plaza areas. Connectivity between the different nodes can thus be leveraged to create the desired quality of the space, including allocation of desired spatial function, and in the process influence the user route choices.

Third, by exploring the movement flow between and within various functional and spatial components of KA, we quantify and reveal a robust 3D circulation. High building circulation is an essential design intent of mixed-use developments, especially in developments like KA, where most functions are distributed across horizontal and vertical dimensions^62^. Our results also show that the spatial function in KA is sufficiently heterogeneous to allow for a large volume of trips with diverse origins and destinations. Vertical streets (stairs, escalators, and elevators) are built to be the backbone of high-rise developments. Hence, it is no surprise that we found these vertical streets to be the hub of the KA spatial network, forming the main pathway of the integrated development. Although observations and theories from previous studies have hinted at some of the inferences drawn here^45^, our study offers a quantitative assessment of the complex relationships between the functional and spatial components of an integrated development through the perspective of human movement flow based on high spatiotemporal data.

One limitation of our work is the relatively small number of participants, due to the constraints of the peer-to-environment sensing that requires the active recruitment of volunteers willing to take part in a long-term study. More passive systems like Wi-Fi sniffers could help track a larger population but inevitably lead to serious privacy concerns. Moreover, the study’s data collection period happened in February 2021, which is a recovery period from one lockdown due to COVID-19 in Singapore. Although most of the daily activities were starting to be resumed during that time, the Singapore Government advised the public to reduce social activities and gatherings. Thus, the human movement patterns in this study could also have been affected by the preventive measures in place due to the COVID-19 pandemic. We have not identified and quantified all the factors that influence user movements, such as the human aspect, spatial aspect, location, time, and weather^19,44^. Hence, more data and analysis are required to fully understand user movement in complex built environments. This also includes constructing co-presence networks of users to study their social interaction and recording user occupancy data to study space utilization. The use of appropriate distance measures (topology, Euclidean, angular, and hybrid) to study spatial networks^48,50^ is an ongoing research. Our study is potentially limited by the use of Euclidean distance and more research is required to understand the impact of using particular distance measures on the phenomenon studied.

Our study does not incorporate design features that can differentiate design classes between developments that can potentially have the same geographic or abstract network representation. This is especially useful when a comparison between different development’s spatial design and user movement is explored. The network-theoretic approach offers the potential to include distinct design features as node or edge properties to make such comparisons. However, more data-driven research is required to test the effectiveness of this approach. In addition to exploring design-dependent properties, population-dependent features can be incorporated into the social network of users that traverse these spaces. This social network can be encoded with their co-presence^63^ (or contact patterns—time and space in which two users are present simultaneously), psychological profile, spatial cognitive profile, and user preferences^64^. The simultaneous study of the interaction between the spatial network of the built environment and the social network of users (known as a socio-spatial study) is a hot topic of research in human mobility studies^65^.

Future cities may need to grow dense and vertical to meet the UN-SDG goals for 2030. Urban planning and design for these cities is tied to our understanding of how people use the built environment. Integrated development is not an independent urban element; on the contrary, it is fully integrated into districts and neighborhoods. This anchoring in a given district/neighborhood has to be taken into account (e.g., mass rapid transit, linkways, bridges). The lack of understanding of human movement in the built environment such as integrated developments can lead to poor designs of related buildings and ineffective integration within the overall urban space. More research is required to expand this study beyond the building to the district scale. Liang and Kang^66^ list four critical challenges for the urban planners and designers to use the insights gained from spatial network analysis: (1) data openness and privacy, (2) lack of direct policy implications, (3) lack of civic, communicative, and collaborative engagement, and finally (4) difficulty to automate visualizations and integrate with Geographic Information System (GIS). Research and practice related to these four challenges are key to the development and use of data-driven planning and design approaches from the building all the way up to the city scale. Only then, future cities can grow vertically and accommodate higher densities while remaining livable.

## Methods

This study uses a data set obtained from a human-tracking experiment carried out at KA for over one month (February 2021) involving 51 distinct participants. This experiment has been designed to collect movement data from regular users of KA as they move around in a free-living environment. The following sections describe the data collection system and processing, movement data, study site, and the derived spatial network.

### Data collection and processing

The data collection system is based on a peer-to-environment Bluetooth localization method that consists of three components: (1) stationary low-energy Bluetooth beacons (Kontakt Smart Beacon Pro BP16-3), (2) custom-built data collection mobile app (iOS and Android), and (3) an access to the AWS cloud server. One hundred twenty-four beacons are placed in locations of interest within and around the KA building perimeter. They transmit their unique ID with a sampling interval of 10 Hz, which is scanned and stored by the participant’s smartphone, and the information is eventually uploaded to the cloud server.

The beacon placement strategy is based on the varying sizes (and signal blocking physical features) of the location grid, which called for the customization of the beacon transmission range (10–50 m). That also allowed us to reduce the signal interference between the beacons and limit the number of beacons detected at each location grid to 1–3 units by design. In turn, this enabled us to implement a straightforward and accurate localization method within the building. This localization is based on a simple transformation table that relates the scanned beacon with the highest Receiver Signal Strength Indicator (RSSI) to a single location grid/zone. To create this table, 4 h of training data are collected using two smartphones (Google Pixel 4a and Samsung Note 8). This ground truth data has been acquired by two researchers carrying these mobile devices while covering systematically the process area of KA, the data being recorded with a sampling rate of 1–0.2 Hz.

The exact same path was recorded twice with the two smartphones. Subsequently, a Naive-Bayes machine learning model is trained on 60% of the training data collected from one mobile and tested on the remaining 40% collected from the second mobile phone. The model achieved a classification performance of 100% accuracy at floor level and 80% accuracy at the node level. Furthermore, it reached a 93% accuracy at a slightly coarser location prediction involving the nearest neighbor. The nearest-neighbor level prediction accuracy of 93% is suitable for our study as our analysis consists of grouping the individual location zones and studying the movement data by aggregation. The transformation table that maps the beacons (with the highest RSSI) to each location grid/zone is thus generated and applied to the mobility data set.

### Description of movement data

**Figure 7:**
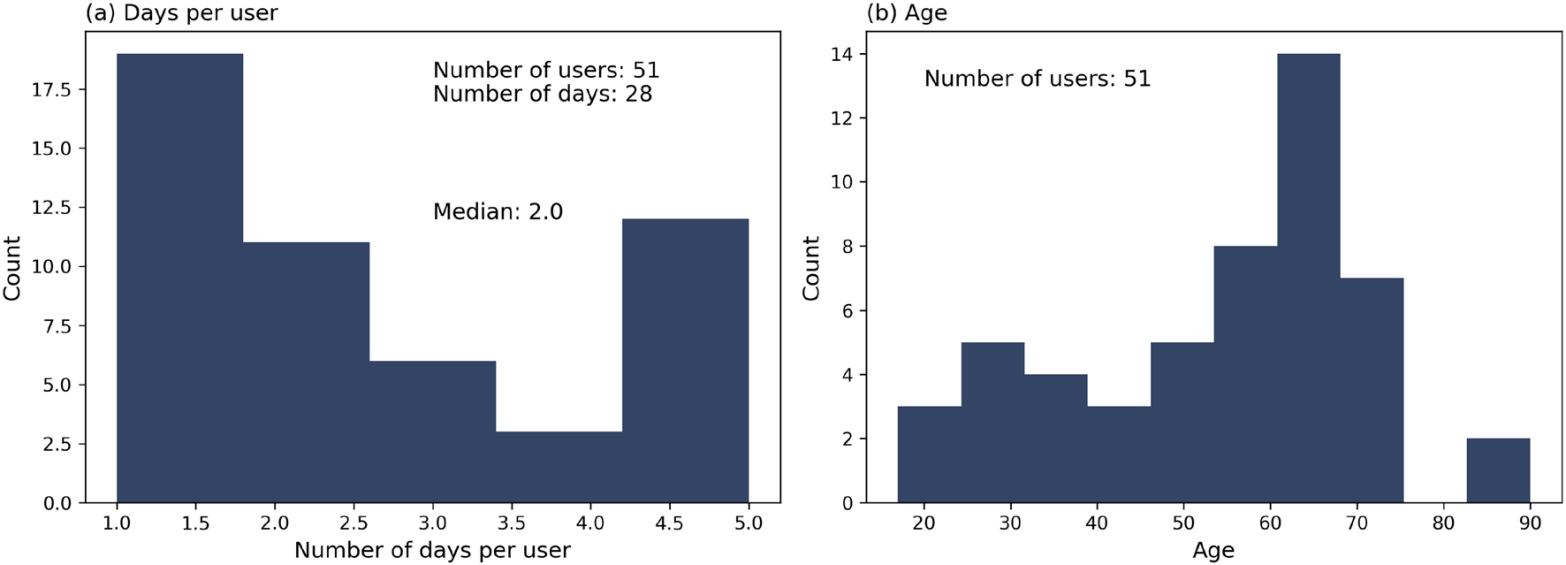
KA Movement data—Histogram of (**a**) number of days per user and (**b**) age.

The data set consists of movement data collected from regular users of the Kampung Admiralty building with duration of recording per participant varying from minimum one day up to a month in February 2021. Volunteers were recruited by representatives in booths or through posters placed around KA. They were offered vouchers worth S$30 in two installments—during recruitment and later at the end of the data collection effort—as an incentive to keep the mobile app installed. Each participant is tracked continuously for the entire duration of the experiment (i.e., day and night). However, their location can only be determined if they are ambulating within KA. To reduce the impact of a few users with long tracking data from dominating the movement data, the maximum number of days per user is restricted to 5 days (refer Fig. 7a for a detailed breakdown of the number of days per participant. The group of 51 participants comprises 21 males and 30 females between the age of 17 up to 90 years old, with 50% below 59 years old (refer Fig. 7b for a detailed breakdown of participant age). The high elderly demographic is representative of KA’s regular users whose residential complex is meant to house seniors and includes community facilities like Active Ageing Hub. Of the 51 participants, 18 are residents, 9 are employed within KA, and 24 are frequent visitors. A total of 131 participating days (i.e., sum of 1-day worth of data for all users) from 51 participants were extracted from the KA data set.

The trip data is derived from the time series of predicted locations using the transformation table. Each trip data is a path through the KA spatial network. This also allows us to fill in missing nodes (location) in a given path by reconstructing it using the shortest path between the known locations for a conservative estimate. The predicted series of nodes in a path has a length of 3 nodes or less for 87% of the missing patches applied. Hence, even though this trip prediction method partly depends on the topology (shortest path calculation), the above numbers suggest that it does not significantly impact our study on the correlation between topology and user movement. A total of 581 paths were derived, of which 361 are continuous in time with a pause time of fewer than 5 min at each stop (i.e., $\le \,5$ min). These 361 paths are defined as a trip in this study, whose origin and destination form the entries of the Origin-Destination matrix. The derived 581 paths encompass 510 paths from weekdays and 71 paths from weekends. A brief comparison between weekdays and weekends is shown in Supplementary Figs. S8 and S9, and Supplementary Tables S5 and S6. It is important to note that the data obtained on weekends is too small to make a meaningful comparison.

A pause of 5 min is selected based on the following consideration: (a) a pause should reflect a destination with sufficient time to perform activities in KA such as visiting a store, or making inquiries in community facilities, (b) allow minimal time for users to move from one location to another, (c) account for wait times in lift lobbies or potentially missed localization. In human mobility research, a pause time of 10–20 minutes is considered typical to define a trip for traveling across cities or large geographic areas^67^. However, KA’s compact space and multiple opportunities for destinations require a relatively smaller pause time. Please refer to Supplementary Figs. S2–S7 and Supplementary Tables S1–S4 for results obtained with a pause time of 10 min. More information on the study site and the KA spatial network is given in the following sections.

### Study site

**Figure 8:**
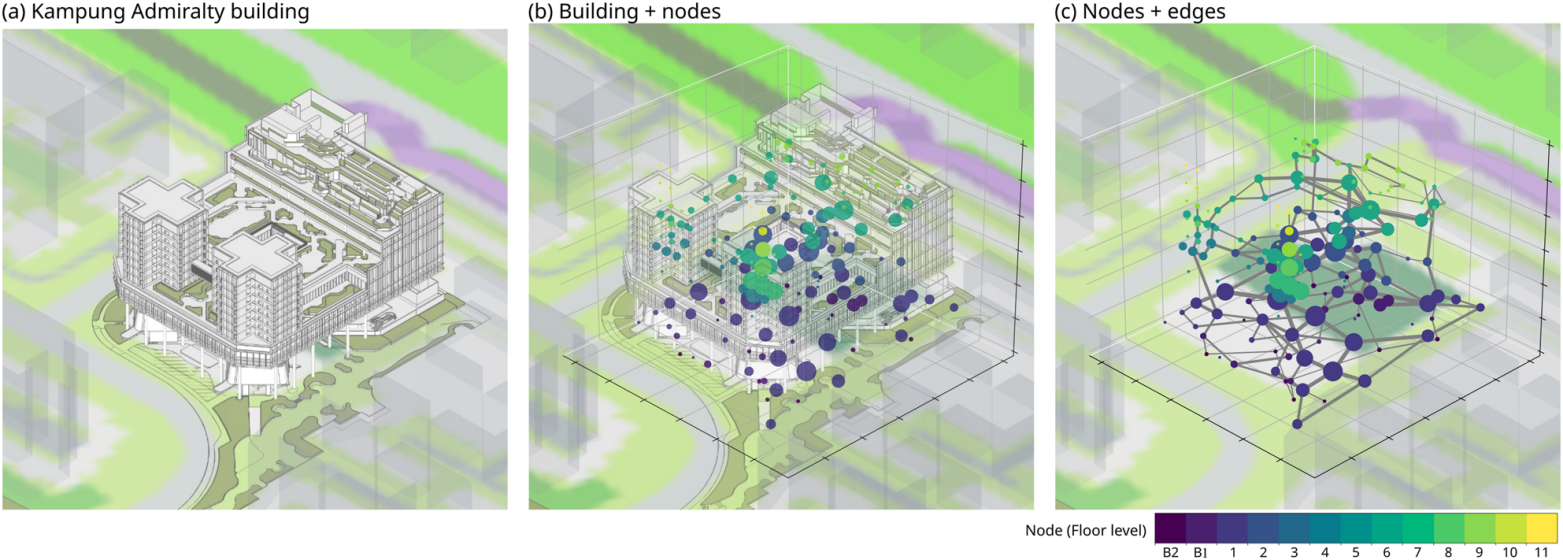
The study site. (**a**) The Kampung Admiralty building, (**b**) building and nodes, where size indicates incoming flows and color indicates floor level, (**c**) nodes and edges (weighted by the sum of flows in both directions).

Kampung Admiralty is Singapore’s first vertically integrated public development (11 stories and 2 levels of basement, completed in May 2017) that brings together a mix of public facilities and services under one roof (see Fig. 8a)^68^. As the increasing urban density in Singapore demands creative ways of intensifying land-use effectively in the vertical dimension, the elevated and layered urban design and architecture approach to the project led to transforming the 0.9 ha site into a dynamic vertically integrated mini-neighborhood for the community. KA’s designers used a ‘sandwich layered’ approach to vertically join various urban functions into that 0.9 ha area. This enhanced the mixed land-use management and living experience of the residents of KA and its vicinity. As an integrated development, the KA is immediately adjacent to a bus stop and a train station (mass rapid transit: MRT station Admiralty). We consider KA to be a high-density development due to the following reasons—(1) population density: KA is placed in a residential zone, the Woodlands East sub-zone, where the population density is very high ($\sim \,38,000$/km^2^), (2) Floor Area Ratio (FAR): KA’s FAR is 3.6, a relatively high ratio for a residential zone, and (3) integration of multiple functions: KA integrates various amenities and services within a relatively compact area, a characteristic of high-density developments, aiming to optimize land use and promote a sense of community.

KA consists of two main parts: the community side and the residential side. The latter comprises two residential towers (the plus/cross shape towers in Fig. 8). The rest of the area forms the community side, which includes multiple types of facilities such as childcare, elderly care, car-park, hospital, shops, bank, and restaurants (refer to Supplementary Fig. S11 for the distribution of these functions across the floors).

### KA spatial network

KA provides a mix of public and common spaces distributed across multiple levels and a mix of spaces with recreational facilities. The whole public space of KA is subdivided into a total of 165 space units (Fig. 8b), and the adjacency connections are linked as shown by the edges (Fig. 8c). A *node* in the KA network is defined as a programmed space with defined boundaries. An *edge* of the network is directed, and it is formed between two reachable adjacent spaces. Thus, the KA spatial network has 165 nodes and 476 edges (Table 5). The routing distance between the centroid of any two nodes is assigned as an edge weight. The KA spatial network consists only of public, open, and freely accessible spaces as nodes. It is primarily built to analyze the relationship between public spaces and their usage based on the participant tracking data. Many closed or private spaces like shops, restaurants, medical facilities, etc., are not considered here as nodes. The exploded axonometric view of KA with functions in each floor and the abstract representation of nodes (and edges) in each floor are shown in Supplementary Figs. S11 and S12 respectively. The nodes are manually determined from the floor plans and the routing distance between them is measured from KA’s floor plans. The network is constructed and analyzed through the NetworkX library in Python^69^.

The aggregated movement flows are shown in Fig. 8b,c, in which the amount of traffic arriving at a node is shown by the node size, and the size of the edges measures the total edge weight irrespective of the flow direction.

**Table 5:** Basic information about KA’s spatial adjacency network, including number of nodes (*N*), number of edges (*E*) within floor-group, and the mobility of nodes (incoming flow) and edge.

Floor-group	*N*	*E*	Total flow	Node’s flow range	Node’s flow mean (std)	Edge’s flow range	Edge’s flow mean (std)
All nodes	165	476	4655	0–154	28.21 (± 37.03)	0–67	9.78 (± 13.47)
Car-parks	25	66	183	0–42	7.32 (± 9.75)	0–24	2.77 (± 5.12)
Level-1	33	102	1450	0–140	43.94 (± 37.27)	0–67	14.22 (± 14.35)
Level-2	19	54	741	0–144	39.00 (± 43.86)	0–64	13.72 (± 15.79)
Level-6*	25	72	878	4–131	35.12 (± 32.25)	0–45	12.19 (± 12.57)
Rooftop garden	19	46	77	0–11	4.05 (± 3.50)	0–6	1.67 (± 1.85)
Residential*	44	88	890	0–129	20.23 (± 32.20)	0–55	10.11 (± 15.62)
Other	9	16	61	0–40	6.78 (± 12.97)	0–40	3.81 (± 10.29)

*Level-6 and residential towers share several nodes on the sixth floor where they are interconnected, including the connection bridge and several public spaces. Category Other contains L3 (1 node), L4 (1 node), and L7 (7 nodes).

### Aggregated spatial networks

The movement data is also studied on an aggregated network. Two types of movement data are aggregated : (a) user movement from one location to its neighboring location in the network (4655 adjacent movements), (b) origin and destination of a trip taken by a user continuously moving with a pause time of less than 5 min in each location (361 trips). By aggregating the data by some combination of floor levels, we preserve the spatial adjacency and aim to study the movement flow across the vertical dimension. The floor-group components of the spatial network is also studied as sub-graphs without aggregation, thus preserving the individual location nodes and used to study within-group movement flows. The movement data is also aggregated by function that ignores the spatial adjacency and used to understand the movement flow between function categories.

#### Aggregation by floor

In this study, we adopted a specific framework to represent the multiple floors of this elevated building. Basements (B1 and B2) are grouped and defined as car-parks. Each of the levels L1, L2, and L6 became a group. Level-1 is the ground floor of KA, which is afforded a large open plaza space, several shops, and some entrances from different directions in the neighborhood. Level-2 is a food court, which can only be accessed by walking from L1 or the community lift connecting to the rest of KA’s community side. Level-6 includes a childcare center, an active ageing hub, a playground, a fitness corner, a garden, and two connections to the social spaces in the residential tower side. Level-6 of the residential towers contains some public social spaces with two connections to the community side of KA that shares the space with the rest of L6. Therefore, the L6 floor and the residential towers share those public social spaces in the network analysis. Level-8 and L9 have a community farm, a community garden, and several sitting places; the two floors were grouped as a rooftop garden. Level-3 and L4 of KA is a medical center, which can be accessed by appointment only, hence containing only one node (lift lobby) for each of these two floors. In L7, the two community facilities (childcare center and active ageing hub) are connected to the lift lobbies, making seven nodes. These three levels (nine nodes) are grouped as ‘other’ and would not be discussed due to their simple structure. Some basic statistics of the network and the six floor groups (car-parks, L1, L2, L6, rooftop garden, and residential) are shown in Table 5.

#### Aggregation by function

The network is also aggregated by function (Table 6). A total of eight function categories are included: (a) entrance (12 nodes), (b) social (15 nodes), (c) commercial (24 nodes), (d) community (18 nodes), (e) garden (23 nodes), (f) residential (14 nodes), (g) vertical (48 nodes), and (h) corridor (11 nodes). The analyses and discussions in this study used both floor and function categories as the grouping methods.

**Table 6:** Spatial functions.

Function	Locations
Entrance	Dropping points and walkways connecting to KA
Social	Seating area, community plaza, fitness corner
Commercial	Restaurant, bank, food court, supermarket, shops
Community	Childcare, elderly care, car-park
Garden	Garden around sitting area, walkways, and roof top gardens
Residential	Apartments
Vertical	Lift, escalator, and stairs lobby
Corridor	Corridor areas not associated with above

### Network measures

To understand the variation of outgoing and incoming flows, the outgoing/incoming entropy is commonly considered^70^:

1 $$\begin{aligned} \mathrm {H^{out}}(c)&= \frac{-\sum _{i=1}^{N} \mathrm {P^{out}}(c,i) \times \log _2 \mathrm {P^{out}}(c,i)}{\log _2 N}, \end{aligned}$$

2 $$\begin{aligned} \mathrm {H^{in}}(c)&= \frac{-\sum _{i=1}^{N} \mathrm {P^{in}}(i,c) \times \log _2 \mathrm {P^{in}}(i,c)}{\log _2 N}, \end{aligned}$$

where $\mathrm {P^{out}}(c,i)$ is the proportion of flow leaving from group *c* to any group *i* divided by the total outflow from group *c*; $\mathrm {P^{in}}(i,c)$ is the proportion of flow coming to group *c* from any group *i* divided by the total inflow to group *c*; *N* is the total number of groups. The term ’group’ here refers to the floor groups or location categories.

### Supplementary Information

Supplementary Information.

## Data Availability

The generated network and relevant materials are available at the following public GitHub repository (https://github.com/ajaymanivannan/Kampung_Admiralty_Dataset_v2), which includes the spatial network file with mobility data, anonymized participant’s trip data, and beacon to zone transformation table. The research involving human subjects and the respective experiment protocol has been reviewed and approved by the Singapore University of Technology and Design (SUTD) Institutional Review Board (IRB) under reference code S-20-353. Informed consent has been obtained from all human participants and the experiment followed the necessary guidelines and regulations concerning research involving human subjects. The supplementary information file is available online along with this article.
