# Supplementary material for: Effects of the interplay between topology and function of an integrated urban development on patterns of user movement
Source: Sci Rep. 2024 Mar 25;14:7021. doi: 10.1038/s41598-024-57475-3 (PMC10963754; doi:10.1038/s41598-024-57475-3)
Supplement: Supplementary file 1 — Supplementary Information. [file 41598_2024_57475_MOESM1_ESM.pdf]

Supplementary Information: Effects of the interplay between topology and function of an integrated urban development on patterns of user movement

Ajaykumar Manivannan[1,+], Wei Chien Benny Chin[2,+], Srilalitha Gopalakrishnan[3], Daniel KH Wong[4], Thomas Schroepfer[3,4], and Roland Bouffanais[5,*]

[1]Department of Mechanical Engineering, University of Ottawa, Ottawa, K1N 6N5, Canada
[2]Department of Geography, National University of Singapore, 117568, Singapore
[3]Future Cities Laboratory Global, Singapore-ETH Centre, 138602, Singapore
[4]Architecture and Sustainable Design, Singapore University of Technology and Design, 487372, Singapore
[5]Department of Computer Science & Global Studies Institute, University of Geneva, CH-1211 Genève, Switzerland
[*]roland.bouffanais@unige.ch
[+]these authors contributed equally to this work

1 Removing Vertical node in OD matrix

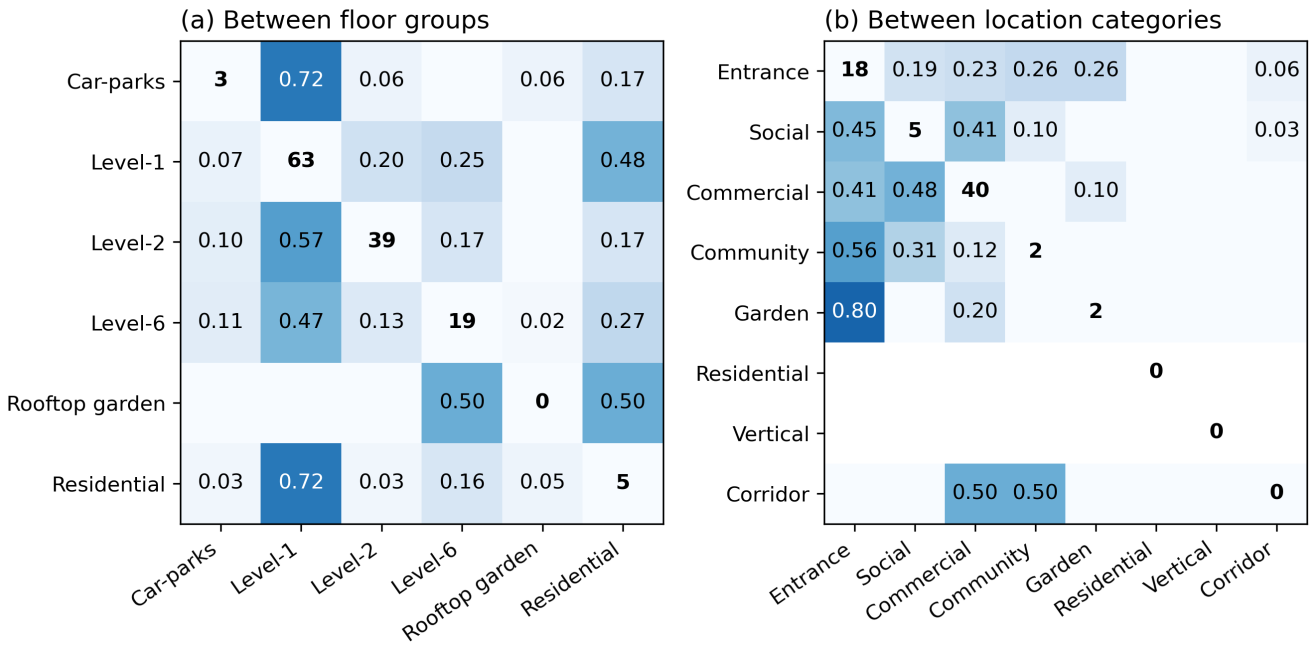

Figure S1. **Row normalized origin-destination matrix — after removing vertical node as origin or destination.** (a) Aggregated floor-groups network, and (b) aggregated function categories network. The row normalized values indicated the proportion of flows from one origin to every destination.

2 Effect of Pause time of 10 minutes

Table S1. Pearson's correlation results between topological network centralities and mobility (incoming flow) for all and each floor-group. The closeness and betweenness centralities are weighted by routing distance. Pause time = 10 minutes.

Floor-groups	In-degree	Closeness (weighted)	Betweenness (weighted)
All nodes	0.466***	0.420***	0.390***
Car-parks	-0.202	0.105	-0.005
Level-1	0.551**	0.420*	0.644***
Level-2	0.632**	0.812***	0.765***
Level-6	0.431*	0.22	0.24
Rooftop garden	0.022	0.504	0.637*
Residential	0.380*	0.364	0.406*

*** : p-value ≤ 0.001; ** : p-value ≤ 0.01; * : p-value ≤ 0.05.

Table S2. The proportion of internal flows and external flows for floor-groups and function categories. 'Residential' in the Floor-group refers to both residential towers, while 'Residential' in the function categories denotes the individual apartments of the two residential towers that exclude vertical transportation and corridors. Pause time = 10 minutes.

Grouping	Category	Total	Internal	External
Floor-group	Car-parks	190	0.75	0.25
Floor-group	Level-1	1479	0.89	0.11
Floor-group	Level-2	679	0.82	0.18
Floor-group	Level-6	571	0.86	0.14
Floor-group	Rooftop garden	44	0.89	0.11
Floor-group	Residential	769	0.89	0.11
Function category	Entrance	372	0.49	0.51
Function category	Social	503	0.34	0.66
Function category	Commercial	1028	0.78	0.22
Function category	Community	284	0.17	0.83
Function category	Garden	233	0.32	0.68
Function category	Residential	36	0	1
Function category	Vertical	1225	0.71	0.29
Function category	Corridor	171	0.01	0.99

Table S3. Normalized outgoing entropy (H^{out}) and incoming entropy (H^{in}) of the floor-group and function categories based on origin and destination of a path. Pause time = 10 minutes.

Floor-group	H^{out}	H^{in}	Function category	H^{out}	H^{in}
Car-parks	0.494	0.526	Entrance	0.722	0.701
Level-1	0.692	0.704	Social	0.582	0.677
Level-2	0.465	0.535	Commercial	0.644	0.612
Level-6	0.688	0.618	Community	0.617	0.569
Rooftop garden	0	0.613	Garden	0.542	0.445
Residential	0.412	0.484	Residential	0	0
			Vertical	0.671	0.609
			Corridor	0	0

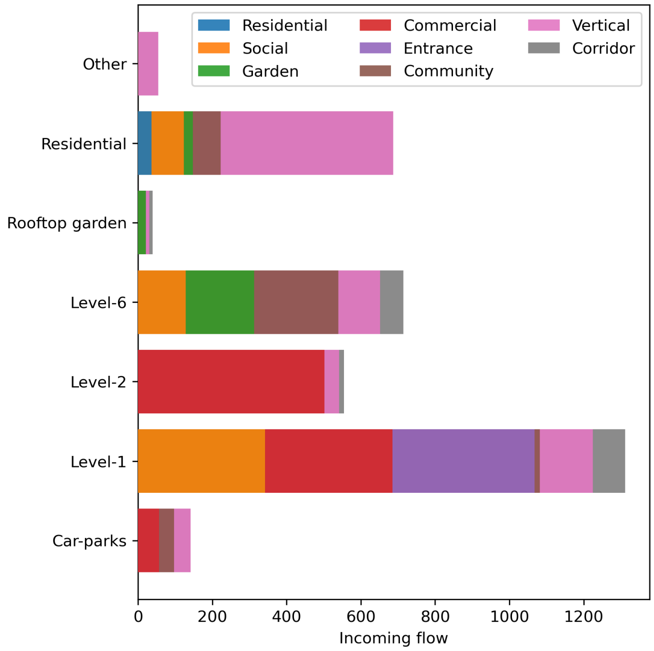

Figure S2. Distribution of inflow by floor-group and program categories. Pause time = 10 minutes.

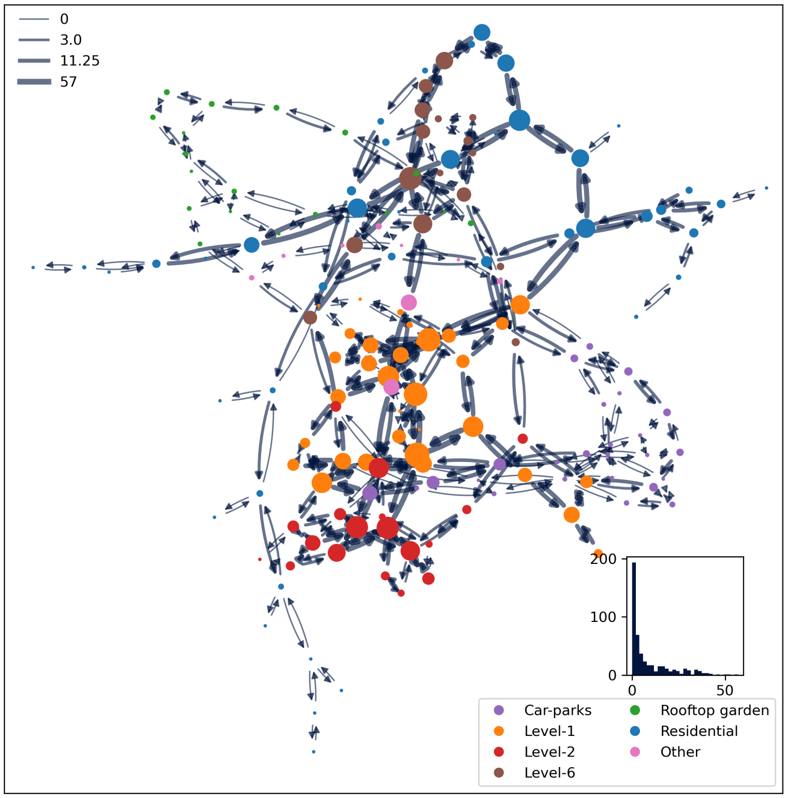

Figure S3. KA spatial network. Nodes are colored by floor group and sized by movement inflow. Directed edges are sized by the number of movement flows in respective directions. Bottom right plot: Histogram of inflow count. Top left: Legend indicating flow size for minimum, 50th percentile, 75th percentile, and maximum values (edges sized by taking the logarithm of flow count for better contrast in visualization). Pause time = 10 minutes.

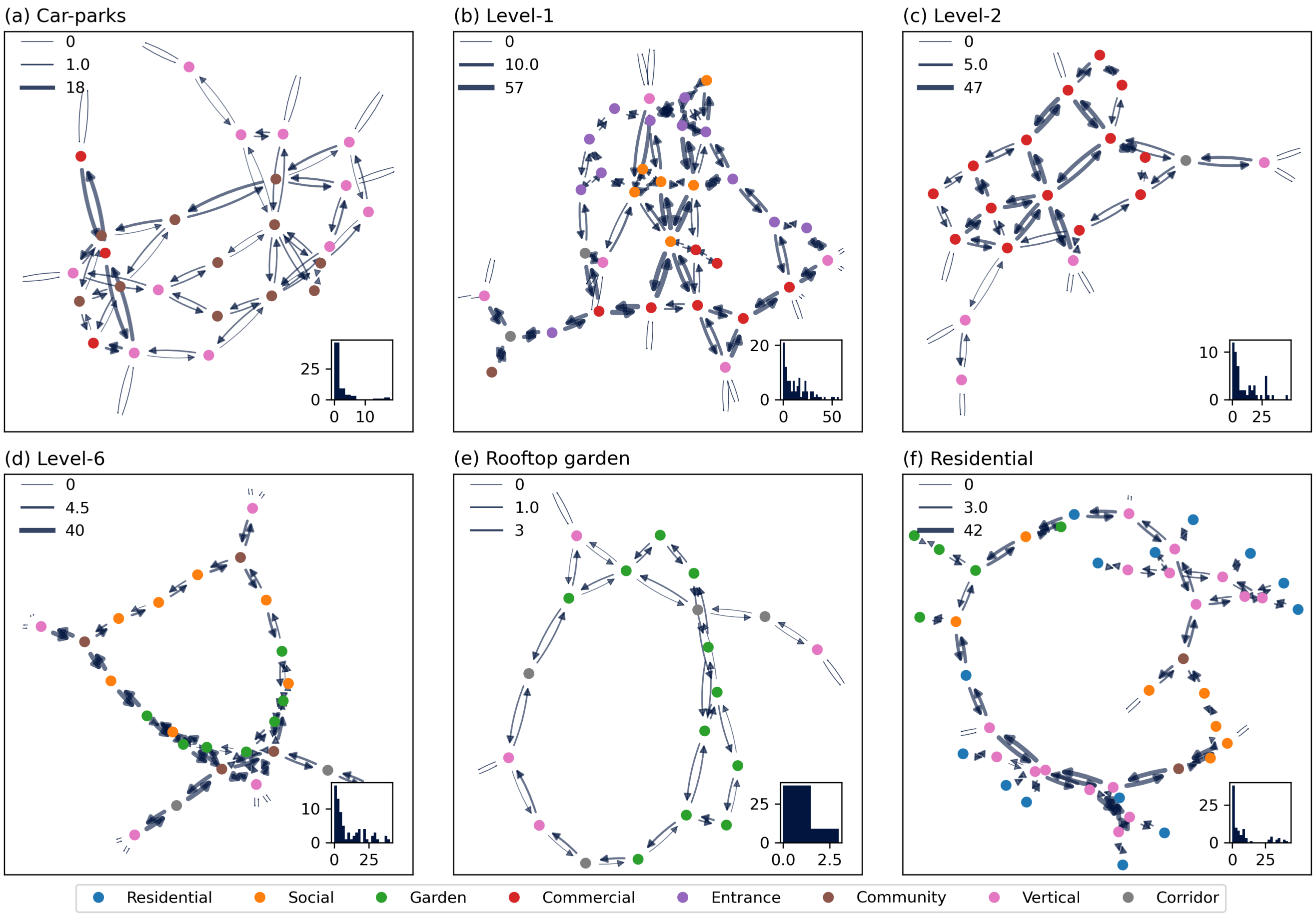

Figure S4. **Subgraphs of the spatial network.** (a) Car-parks (B1 and B2), (b) L1, (c) L2 (food court), (d) L6, (e) rooftop garden (L8 and L9), and (f) the two residential towers. The colors indicates program categories. Bottom right plot: Histogram of flow count. Top left: Legend indicating flow size for minimum, 50th percentile, and maximum values (edges sized by taking the logarithm of flow count for better contrast in visualization). Pause time = 10 minutes.

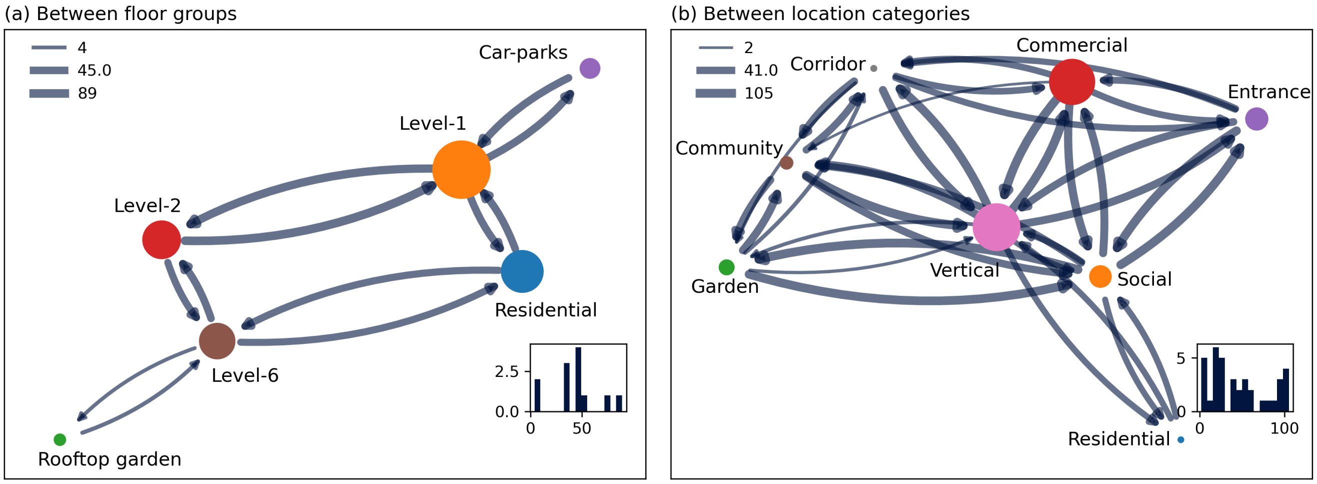

Figure S5. **Networks of aggregated flow.** (a) By floor-groups and (b) by function categories. The node sizes indicate internal flows (self-loop). Bottom right plot: Histogram of inflow count. Top left: Legend indicating flow size for minimum, 50th percentile, and maximum values (edges sized by taking the logarithm of flow count for better contrast in visualization). Pause time = 10 minutes.

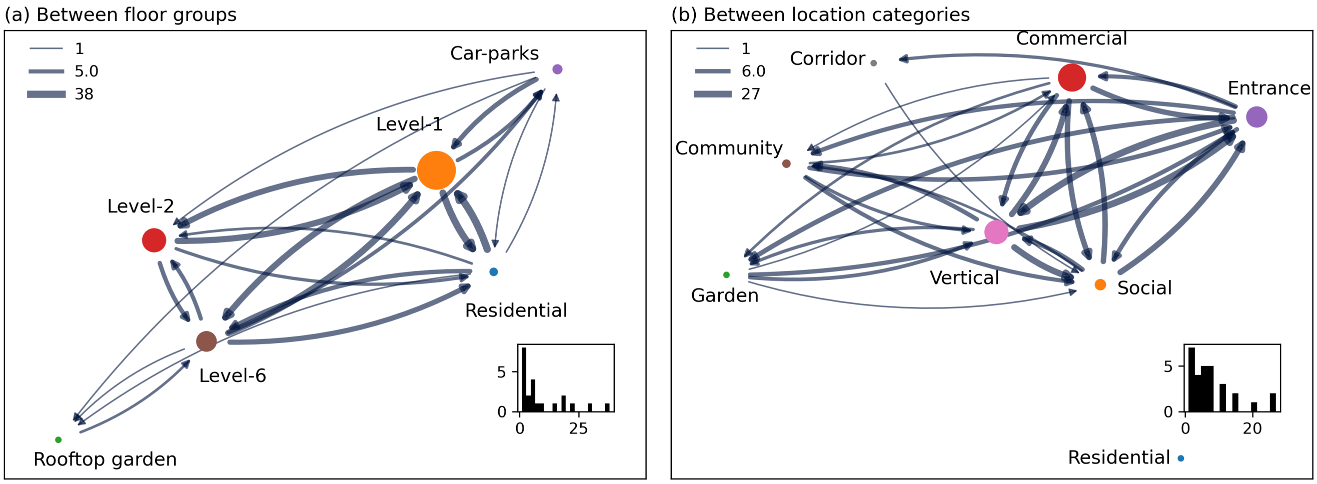

Figure S6. Networks of aggregated flow based on origin and destination of a path (a) By floor-groups and (b) by function categories. The node sizes indicate internal flows (self-loop). Bottom right plot: Histogram of inflow count. Top left: Legend indicating flow size for minimum, 50th percentile, and maximum values (edges sized by taking the logarithm of flow count for better contrast in visualization). Pause time = 10 minutes.

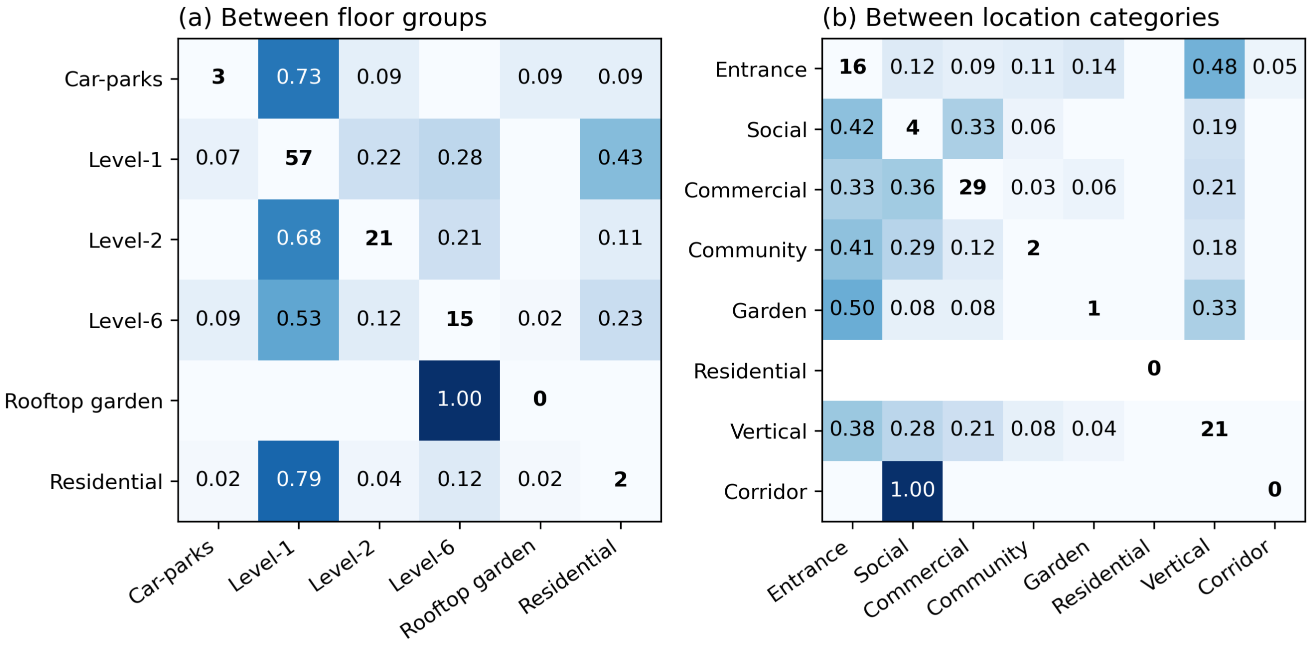

Figure S7. Row normalized origin-destination matrix. (a) Aggregated floor-groups network, and (b) aggregated function categories network. The row normalized values indicated the proportion of flows from one origin to every destination. Pause time = 10 minutes.

Table S4. **Basic information about KA's spatial adjacency network, including number of nodes (N), number of edges (E) within floor-group, and the mobility of nodes (incoming flow) and edge. Pause time = 10 minutes.**

Floor-group	N	E	Total flow	Node's flow range	Node's flow mean (std)	Edge's flow range	Edge's flow mean (std)
All nodes	165	476	3852	0–133	23.35 (±30.67)	0–57	8.09 (±11.19)
Car-parks	25	66	142	0–32	5.68 (±7.35)	0–18	2.15 (±3.89)
Level-1	33	102	1312	0–120	39.76 (±33.39)	0–57	12.86 (±12.87)
Level-2	19	54	555	0–103	29.21 (±30.65)	0–47	10.28 (±11.07)
Level-6*	25	72	715	4–110	28.60 (±26.89)	0–40	9.93 (±10.75)
Rooftop garden	19	46	39	0–4	2.05 (±1.57)	0–3	0.85 (±0.98)
Residential*	44	88	687	0–98	15.61 (±24.13)	0–42	7.81 (±11.76)
Other	9	16	55	0 – 37	6.11 (±11.84)	0 – 37	3.44 (±9.38)

*Level-6 and residential towers share several nodes at the sixth floor where they are interconnected, including the connection bridge and several public spaces. Category Other contains L3 (1 node), L4 (1 node), and L7 (7 nodes).

3 Weekday Vs Weekend

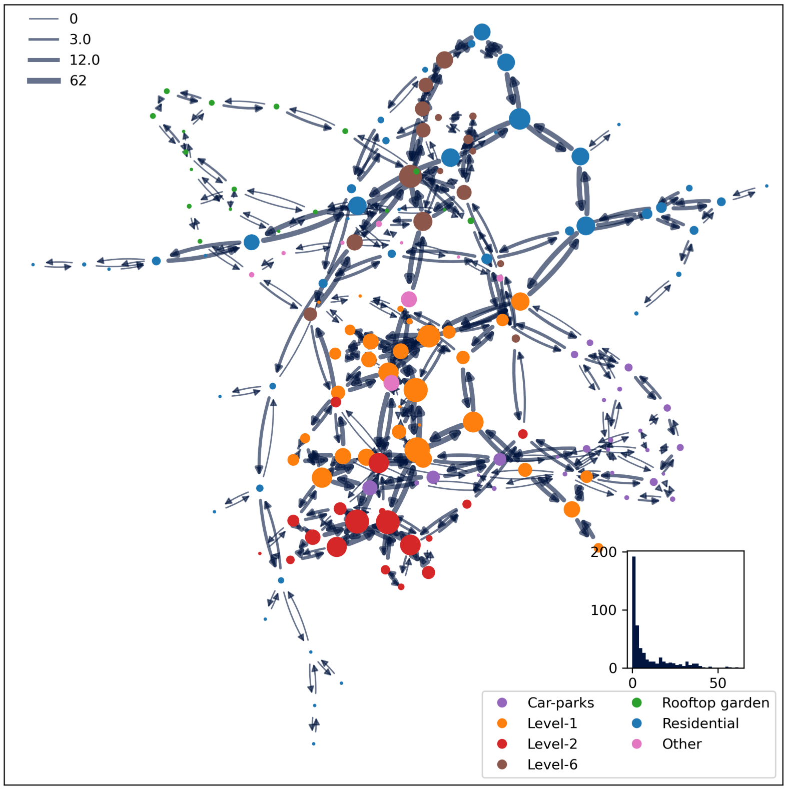

Figure S8. KA spatial network—Weekday. Nodes are colored by floor group and sized by movement inflow. Directed edges are sized by the number of movement flows in respective directions. Bottom right plot: Histogram of inflow count. Top left: Legend indicating flow size for minimum, 50th percentile, 75th percentile, and maximum values (edges sized by taking the logarithm of flow count for better contrast in visualization).

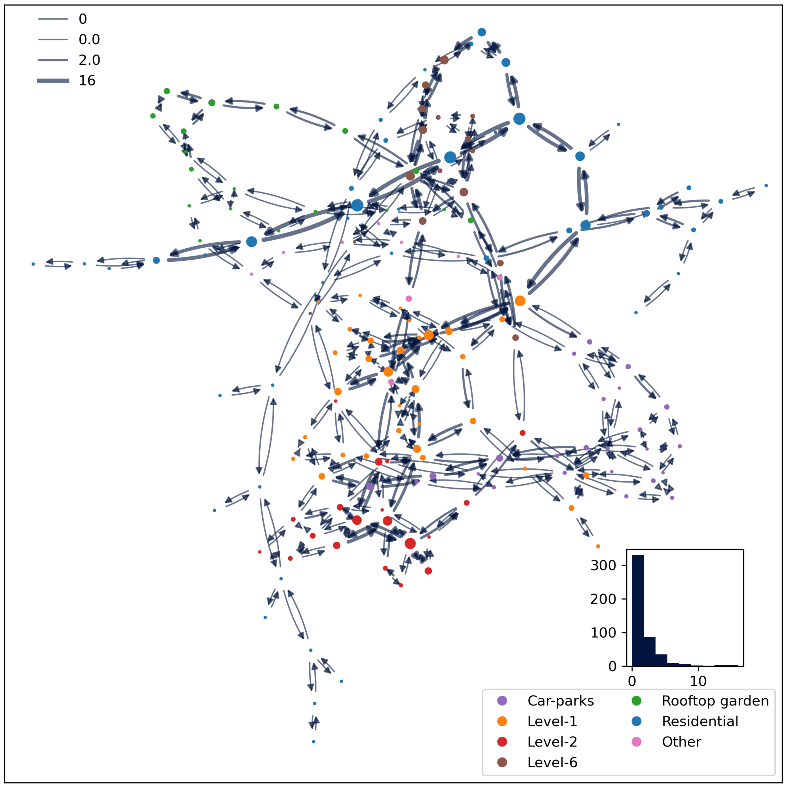

Figure S9. **KA spatial network—Weekend.** Nodes are colored by floor group and sized by movement inflow. Directed edges are sized by the number of movement flows in respective directions. Bottom right plot: Histogram of inflow count. Top left: Legend indicating flow size for minimum, 50th percentile, 75th percentile, and maximum values (edges sized by taking the logarithm of flow count for better contrast in visualization).

Table S5. **Weekday: Basic information about KA's spatial adjacency network, including the number of nodes (N), number of edges (E) within floor-group, and the mobility of nodes (incoming flow) and edge.**

Floor-group	N	E	Total flow	Node's flow range	Node's flow mean (std)	Edge's flow range	Edge's flow mean (std)
All nodes	165	476	3987	0–144	24.16 (±32.35)	0–62	8.38 (±11.70)
Car-parks	25	66	144	0–35	5.76 (±8.15)	0–20	2.18 (±4.27)
Level-1	33	102	1318	0–130	39.94 (±33.75)	0–62	12.92 (±13.17)
Level-2	19	54	645	0–128	33.95 (±38.38)	0–57	11.94 (±13.84)
Level-6*	25	72	747	4–117	29.88 (±28.34)	0–41	10.38 (±11.18)
Rooftop garden	19	46	42	0–5	2.21 (±1.73)	0–4	0.91 (±1.06)
Residential*	44	88	704	0–101	16.00 (±24.51)	0–39	8.00 (±11.84)
Other	9	16	56	0 – 37	6.22 (±11.92)	0 – 37	3.50 (±9.46)

*Level-6 and residential towers share several nodes on the sixth floor where they are interconnected, including the connection bridge and several public spaces. Category Other contains L3 (1 node), L4 (1 node), and L7 (7 nodes).

Table S6. **Weekend: Basic information about KA's spatial adjacency network, including the number of nodes (N), number of edges (E) within floor-group, and the mobility of nodes (incoming flow) and edge.**

Floor-group	N	E	Total flow	Node's flow range	Node's flow mean (std)	Edge's flow range	Edge's flow mean (std)
All nodes	165	476	668	0–29	4.05 (±5.89)	0–16	1.40 (±2.35)
Car-parks	25	66	39	0–7	1.56 (±1.70)	0–4	0.59 (±0.95)
Level-1	33	102	132	0–18	4.00 (±4.56)	0–7	1.29 (±1.70)
Level-2	19	54	96	0–23	5.05 (±6.09)	0–8	1.78 (±2.30)
Level-6*	25	72	131	0–14	5.24 (±4.71)	0–7	1.82 (±1.97)
Rooftop garden	19	46	35	0–7	1.84 (±2.18)	0–3	0.76 (±1.03)
Residential*	44	88	186	0–29	4.23 (±8.00)	0–16	2.11 (±3.99)
Other	9	16	5	0 – 3	0.56 (±1.07)	0 – 3	0.31 (±0.85)

*Level-6 and residential towers share several nodes at the sixth floor where they are interconnected, including the connection bridge and several public spaces. Category Other contains L3 (1 node), L4 (1 node), and L7 (7 nodes).

4 Similarity in mobility flow between different days of a user

The *cosine similarity* for each node i, which measures the similarity between this node and its neighborhoods in the two networks. If $w_{ij,1}$ and $w_{ij,2}$ denote the weights on the links from i to j respectively in networks 1 and 2, the cosine similarity of i is defined as

$$\text{sim}_{1,2}(i) = \frac{\sum_j w_{ij,1} w_{ij,2}}{\sqrt{\sum_j w_{ij,1}^2}\sqrt{\sum_j w_{ij,2}^2}}. \tag{1}$$

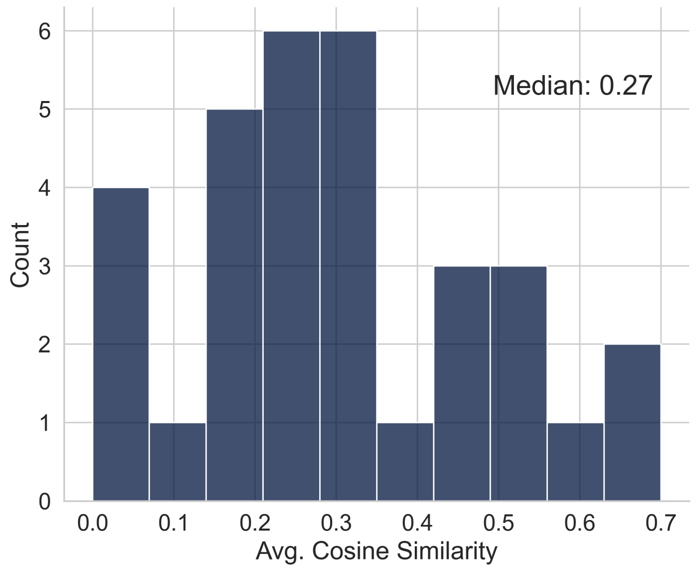

Figure S10. Histogram of Average Cosine similarity between 1-day aggregated networks. It is calculated for different days of a user for all users. Average Cosine Similarity (CS) for user i = (Sum of CS of all pairs of 1-day network for user i)/number of pairs of 1-day network for user i

5 Floor plan to KA spatial network

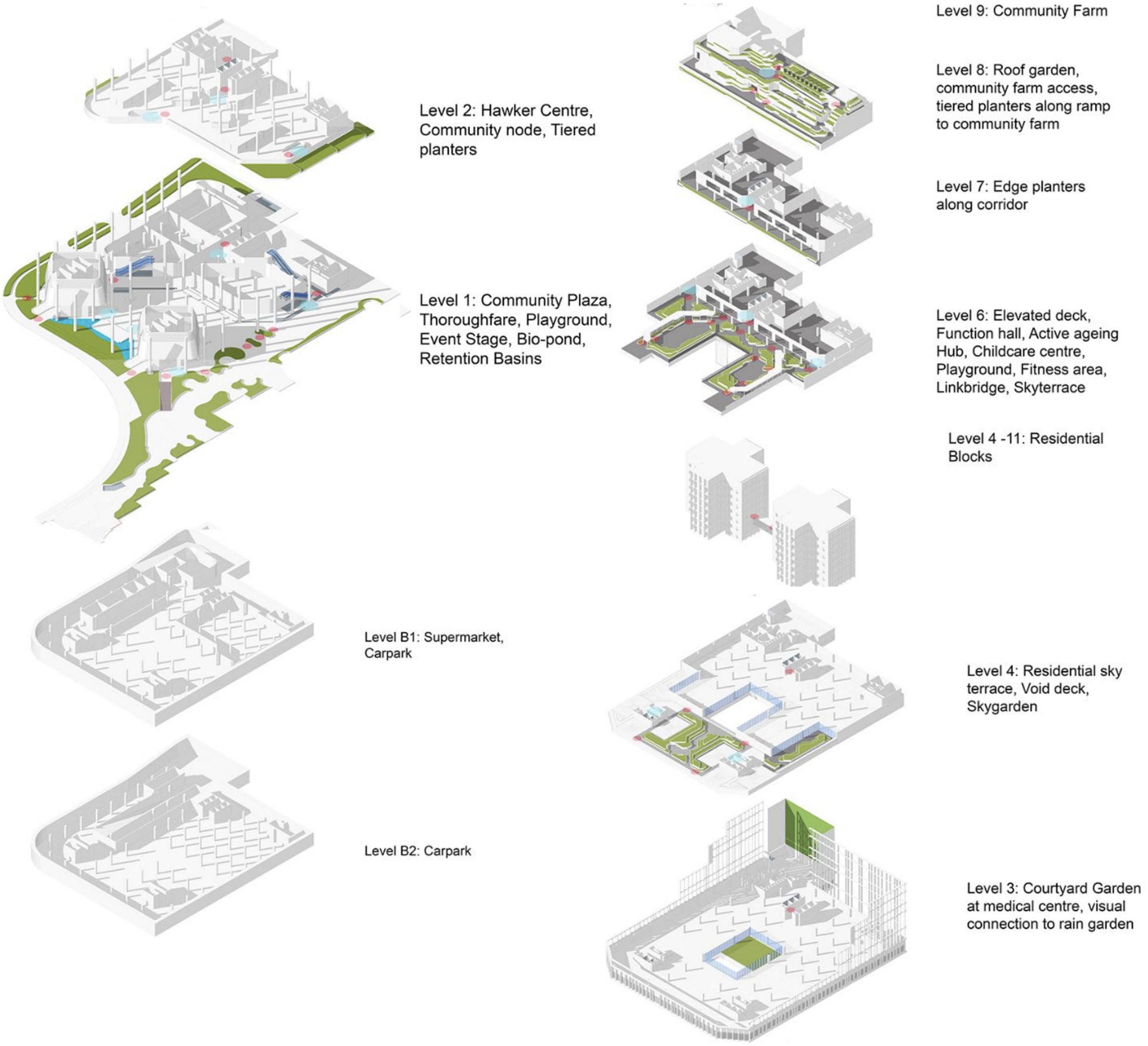

Figure S11. **Exploded Axonometric of Kampung Admiralty.**

Figure S12. Abstract representation of floor plan to network transformation by each floor. The number near the edge represents the routing distance in meters between nodes. The distance is calculated from the floor plans provided by the Architects of KA. Nodes from each floor are stacked on top of each other and connected through vertical facilities like lifts, stairs, and escalators.

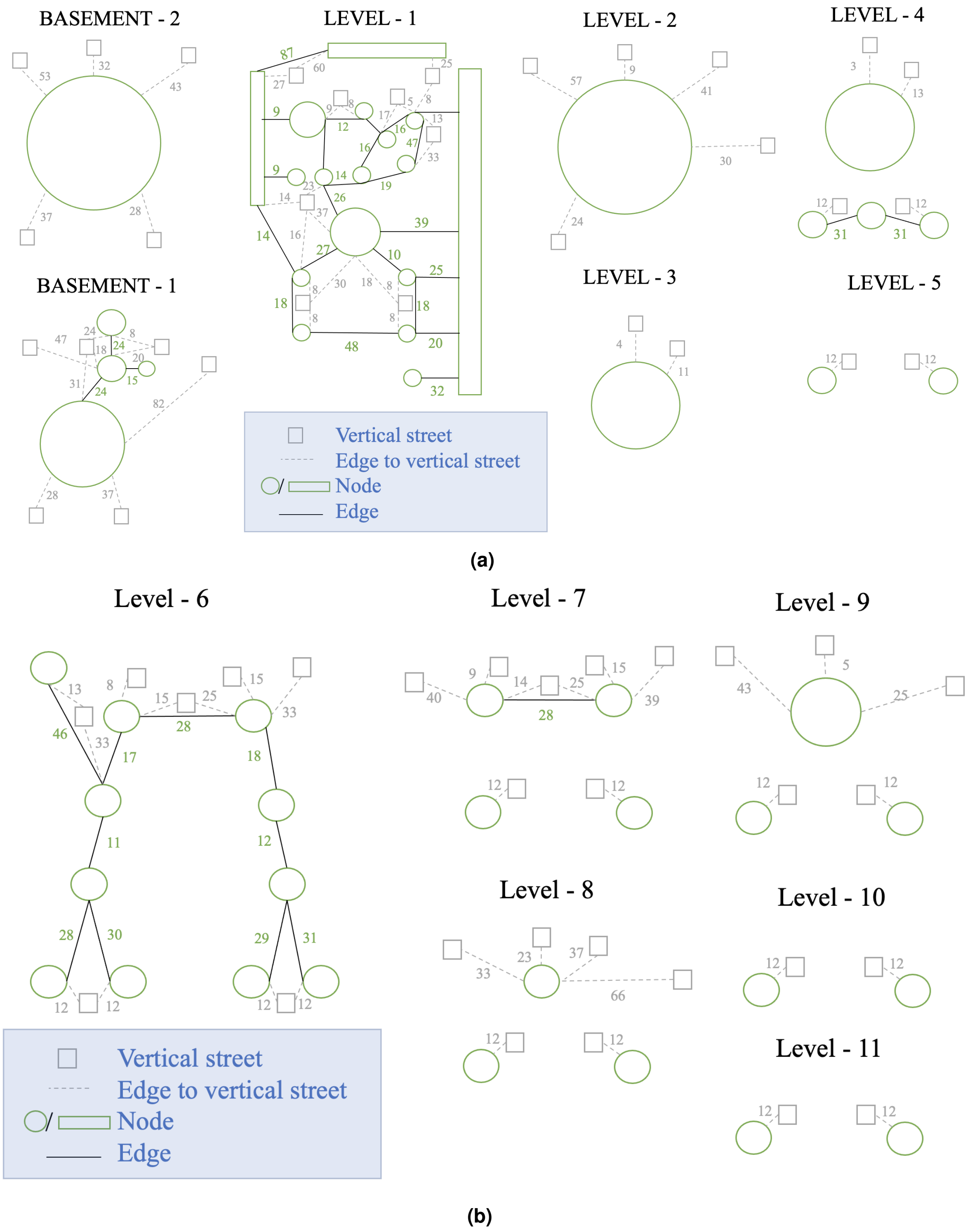

6 Nodes in KA spatial network

A node in the network is an approximate and variable area of space in the KA building that serves a particular function. For example, a walkway followed by an entrance, and a lift area will be divided into three nodes Walkway, Entrance, and Lift (Figure S13). An exception to this case is when the space is large enough that it requires sub-divisions for more accurate tracking of a user along the floor. For example, in Level 1, the Community Plaza (Figure S13) is divided into four nodes (e.g., CP_Zone_N where $N = 1$, 2, 3, and 4). This allows us to calculate routing distance more accurately along the spatial network. A routing distance is the distance between the centroids of the two connected nodes.

The beacon locations are not always related to a particular node. While we make all steps to confine a beacon to capture one particular node (by adjusting the transmission range and orientation of the beacon), this is not always possible or accurate. For these reasons, we used Machine Learning models to relate beacons to nodes based on the trained data that contains records (Beacon signature) of beacons when passing through each node.

There are 165 nodes and 124 beacons. The disparity between the number of nodes and beacons is because many nodes (such as corridors or walkways) do not require a dedicated beacon, as they may serve as an interconnecting node between two other nodes that are tracked by a beacon. Hence not all nodes require a beacon and its location in a user's path is implicitly assumed.

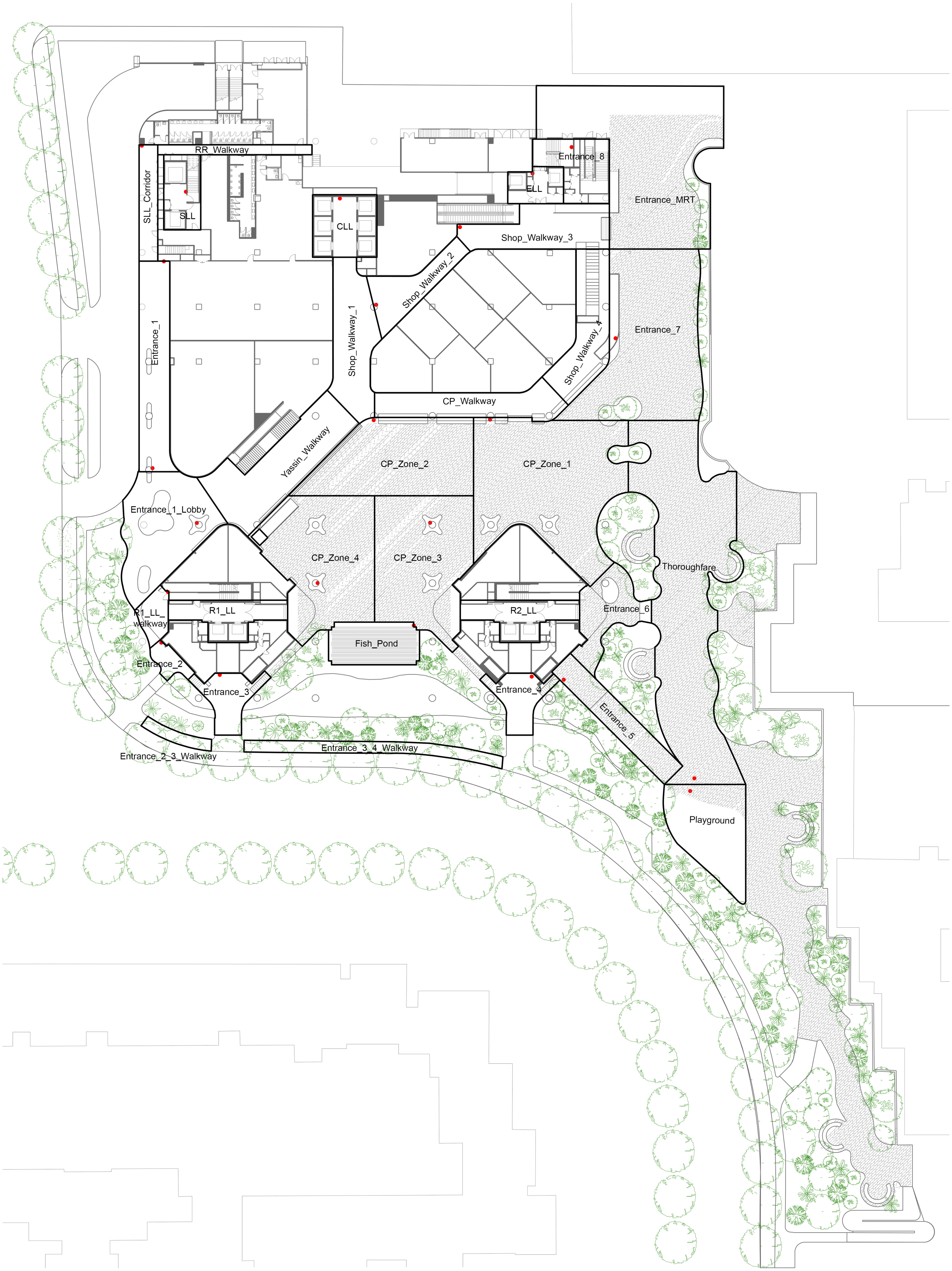

Figure S13. KA Level 1 node divisions

7 Demographic details based on mobility data

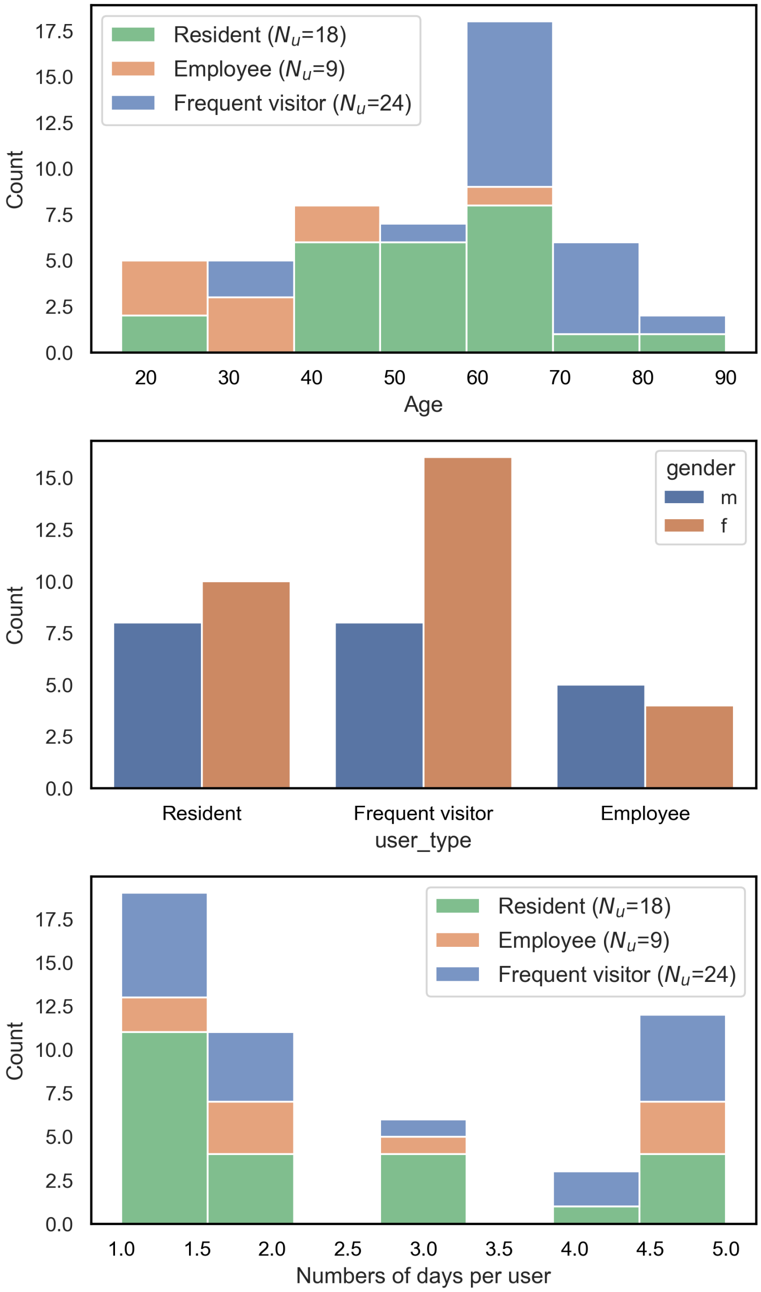

Figure S14. Demographic details by category of participants. Number of users per (a) age, (b) gender, and (c) number of days
